# Multifunctional Scaffolds Based on Emulsion and Coaxial Electrospinning Incorporation of Hydroxyapatite for Bone Tissue Regeneration

**DOI:** 10.3390/ijms232315016

**Published:** 2022-11-30

**Authors:** Amirmajid Kadkhodaie Elyaderani, María del Carmen De Lama-Odría, Luis J. del Valle, Jordi Puiggalí

**Affiliations:** 1 Departament d’Enginyeria Química, Universitat Politècnica de Catalunya, Escola d’Enginyeria de Barcelona Est-EEBE, 08019 Barcelona, Spain; 2 Barcelona Research Center for Multiscale Science and Engineering, Universitat Politècnica de Catalunya, Escola d’Enginyeria de Barcelona Est-EEBE, 08019 Barcelona, Spain; 3 Institute for Bioengineering of Catalonia (IBEC), The Barcelona Institute of Science and Technology (BIST), Carrer Baldiri i Reixac 11-15, 08028 Barcelona, Spain

**Keywords:** multifunctional scaffolds, tissue regeneration, bone tissue, hydroxyapatite, coaxial electrospinning, emulsion electrospinning

## Abstract

Tissue engineering is nowadays a powerful tool to restore damaged tissues and recover their normal functionality. Advantages over other current methods are well established, although a continuous evolution is still necessary to improve the final performance and the range of applications. Trends are nowadays focused on the development of multifunctional scaffolds with hierarchical structures and the capability to render a sustained delivery of bioactive molecules under an appropriate stimulus. Nanocomposites incorporating hydroxyapatite nanoparticles (HAp NPs) have a predominant role in bone tissue regeneration due to their high capacity to enhance osteoinduction, osteoconduction, and osteointegration, as well as their encapsulation efficiency and protection capability of bioactive agents. Selection of appropriated polymeric matrices is fundamental and consequently great efforts have been invested to increase the range of properties of available materials through copolymerization, blending, or combining structures constituted by different materials. Scaffolds can be obtained from different processes that differ in characteristics, such as texture or porosity. Probably, electrospinning has the greater relevance, since the obtained nanofiber membranes have a great similarity with the extracellular matrix and, in addition, they can easily incorporate functional and bioactive compounds. Coaxial and emulsion electrospinning processes appear ideal to generate complex systems able to incorporate highly different agents. The present review is mainly focused on the recent works performed with Hap-loaded scaffolds having at least one structural layer composed of core/shell nanofibers.

## 1. Introduction

**Figure 1:**
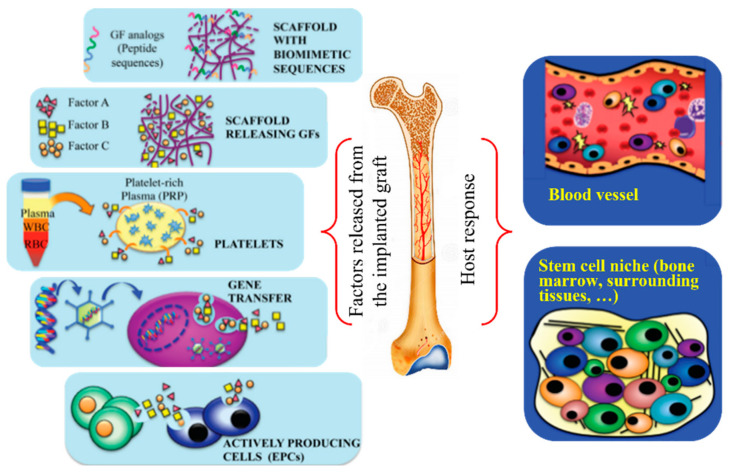
Scheme showing features from implants at the bone defect site that influence the host response. These may include growth factors (or their analogues) released from scaffolds or platelet-enriched plasma-seeded materials, or growth factors released from natural or genetically modified housing cells. In response, cell homing, enhanced vascularization, and bone regeneration will occur. Adapted from [4].

Organ failure and tissue loss are problems that affect millions of individuals annually. It should be taken into account the multiple surgical interventions and the corresponding health care costs that are required to address these issues. In addition, surgical strategies such as organ transplantation, tissue transfer (i.e., from healthy to damaged sites), and even substitution with mechanical devices have serious limitations (e.g., the number of potential organ donors, imperfect matching between tissues, and limited durability of devices). Therefore, tissue engineering appears as a fundamental tool to restore damaged tissue (e.g., by trauma or acquired diseases) and recover its normal functionality. Regenerative medicine refers specifically to the science focused on replacing cells, tissues, or organs and restoring the corresponding biological function [1]. The development of natural or synthetic materials able to improve damaged tissue is a crucial point of tissue engineering [2]. From a biological point of view, it should be considered the capacity of tissues to be constantly remodeled, the possibility of reorganizing cells under appropriate culture conditions, and the advantages provided by a biodegradable and biocompatible template (scaffold) to guide cell reconstruction (i.e., the replacement and rebuilding of the damaged tissue). Obviously, oxygen and nutrient exchange can limit the volume of the implanted scaffold. Thus, final solutions should consider the necessity to provide sufficient vascularization in order to satisfy the needs of nutrient supply and clearance of products. Tissue engineering includes the study of the regeneration of different types of tissues (e.g., urologic, cardiovascular, nerves, skin, cartilage, or bones). Namely, tissues have different intrinsic characteristics that usually require different solutions. Bone regeneration is a complex process that involves an interplay of molecular events that promote the migration, proliferation, and differentiation of mesenchymal cells. For a better understanding of the complexity of the host cellular response to the implantation of bone regeneration scaffolds, Figure 1 is presented [3,4]. Despite the intensive research and the great advances that have been made in the identification and understanding of the cellular and molecular events triggered by the implanted scaffolds, (e.g., the role played by cell-signaling molecules), the activated mechanisms are not fully elucidated and wide room for improvement exists. In addition to the biological aspect that can condition the design and material selection for bone regenerative therapy, there is an important economical factor to be considered, as bone defect repairing represents a cost higher than USD 5 billion annually [4].

**Figure 2:**
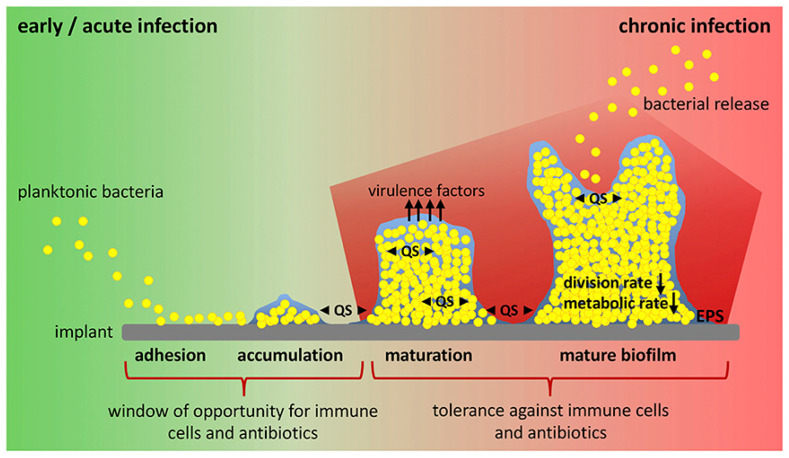
Scheme showing biofilm formation and window of opportunity for effective clearance of bacteria. Biofilm formation leads to an increased tolerance against the immune system, antibiotics, and possible chronicity of the infection. QS and EPS indicate quorum sensing intercellular signalling molecules and extracellular polymer substances, respectively. Reproduced with permission from [6].

In addition, implant-related bone infections are a major problem in orthopedic surgery. Thus, the infection risk through open wounds and broken bones can vary from 10% to 50%, depending on the specific case. This feature constitutes nowadays a serious burden in health care [5]. Biofilm formation over a medical device can easily start with the adhesion of planktonic bacteria through interactions (e.g., hydrophobic, electrostatic, and van der Waals) that enable unspecific attachment (Figure 2) [6]. After this colonization, a biofilm able to shield bacteria from the immune cells is formed. Incorporation of effective antibacterial agents in implants and scaffolds remains an unsolved problem. Polymers offer great potential as matrices for scaffolds and implants since they can be chemically and structurally modified, allowing the modulation of their final properties. Intensive research has been focused on the development of new polymeric-based systems having the appropriate functionalities. In this sense, a great variety of potential new biomaterials are being investigated using high-yield methods and rapid nanoscale synthesis [7]. Chip-like devices can subsequently be employed to screen the cell-polymer interactions that can be established. The impact of structural modifications on the polymer structure can be revealed according to the observed differences in the cellular responses.

Osteoinductive materials have the ability to induce in vivo bone formation through appropriate instructions to their surrounding environment. Natural ceramics (e.g., hydroxyapatite and HAp) and their composites with biodegradable polymers are extensively studied. In general, hybrid materials, blends, copolymers, and composites are preferable to satisfy a wide range of requirements (e.g., mechanical properties, bioactivity, and hydrophilicity). Hydrogels are also interesting systems due to their capacity to mimic the extracellular matrix (ECM) and deliver bioactive agents. Efforts are also focused on the design of materials with the capacity to modulate the immune system and favor bone regeneration. In this sense, surface treatments (i.e., the development of hydrophilic surfaces, anti-fouling coatings, and micro/nano surface texturization) and the encapsulation/delivery of bioactive molecules should be considered.

**Figure 3:**
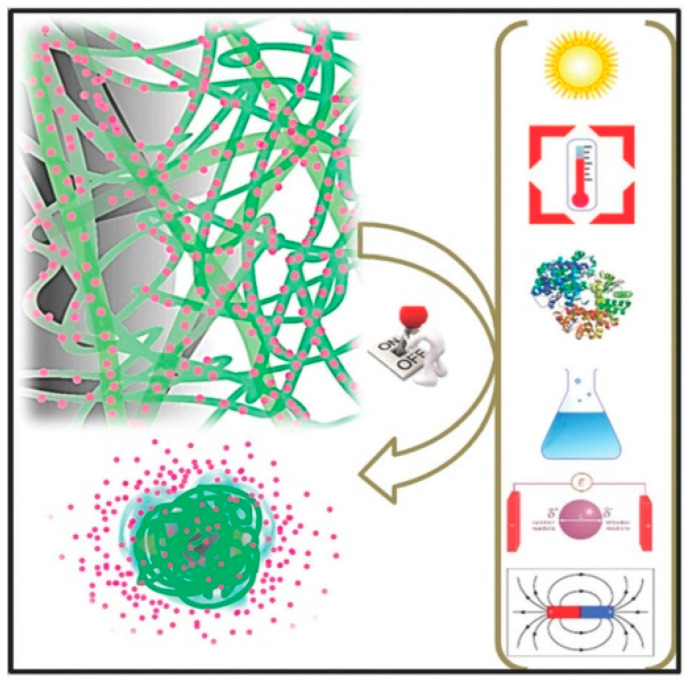
Scheme showing the triggered drug release under biological, chemical, light, temperature, magnetic, and electric field stimuli. Reproduced with permission from [10].

The development of multifunctional scaffolds appears as an essential strategy to fulfill all of the above-indicated requirements. Furthermore, it should be pointed out that therapies for bone cancer (osteosarcoma), such as surgical intervention or radiotherapy, are limited and do not assure the complete eradication of malignant cells. In addition, treatments fully based on a systemic administration of chemotherapeutic drugs are problematic due to the poor diffusion of the active compounds through bone tissues. A high dosage is required with the associated detrimental effects on the organs of the body [8]. The local drug delivery from appropriate and multifunctional scaffolds may facilitate the application of new therapies, such as hyperthermia and photothermal treatments (Figure 3) [9,10].

**Figure 4:**
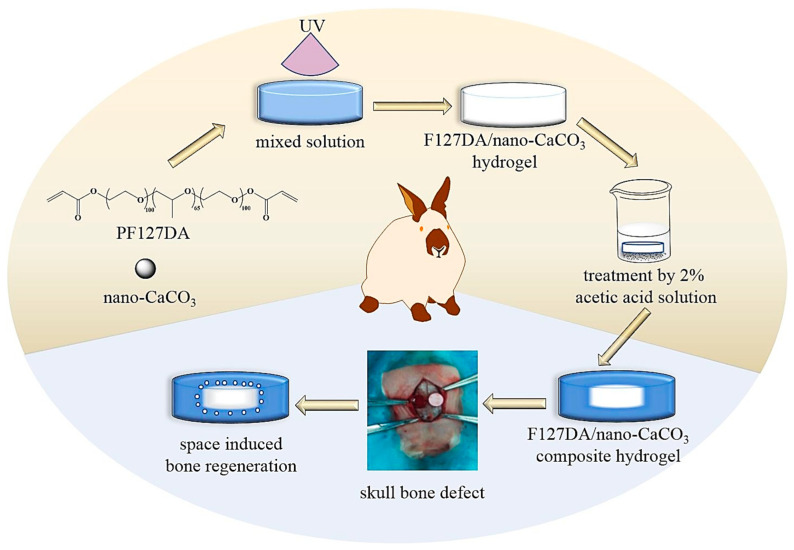
Scheme of a CaCO_3_-loaded hydrogel with triggered Ca^2+^ release under exposure to acid media. Reproduced with permission from [11].

Different preparation methods have been developed to obtain multifunctional scaffolds for tissue regeneration. In general, the most interesting devices are based on complex systems that provide different characteristics, for example, a combination of large and small pores or a distribution of materials with a different affinity with the loaded active agents. Membranes constituted by nanofibers appear interesting as one of the hierarchical constituents since they are ideal to mimic the characteristics of the natural ECM. As aforementioned, hydrogel scaffolds attract attention in this matter since recent developments allow control of mechanical properties while supplying calcium for bone regeneration applications [11]. Specifically, CaCO_3_ nanoparticles can be incorporated into a hydrogel, giving rise to enhanced mechanical properties and calcium release after exposure to weak acid environments (Figure 4). Biomimetic scaffolds based on natural gel-like polymers are highly interesting for bone regeneration due to probed biological safety and the easy ability to modify their surface [12]. Nowadays, electrospinning is a relatively simple technique having an inexpensive setup. The technique can provide porous structural supports for cells and also load them with multiple bioactive factors. Great efforts have been focused on the production of submicron-sized nonwoven fibrous scaffolds, being justified by a detailed discussion of such systems.

Specifically, the present review is focused on the more recent developments concerning multifunctional hybrid scaffolds that incorporate HAp as an osteogenic component and that at least have a layer/membrane fabricated by advanced electrospinning procedures (i.e., coaxial, emulsion, or melt electrospinning). The review is structured in the following chapters: a general overview of HAp characteristics (Section 2); a general discussion about bone tissue strategies and multifunctional scaffolds (Section 3), which includes a description of the most employed natural and synthetic materials for bone tissue regeneration (Section 3.1), surface functionalization (Section 3.2), and incorporation of functional compounds (Section 3.3); a detailed explanation of electrospinning (Section 4) with especial emphasis to coaxial (Section 4.1) and emulsion (Section 4.2) processes; and, finally, detailed exposure of the most relevant advances on electrospun multifunctional scaffolds developed for bone tissue regeneration (Section 5).

## 2. Hydroxyapatite for Tissue Regeneration and Drug Delivery

**Figure 5:**
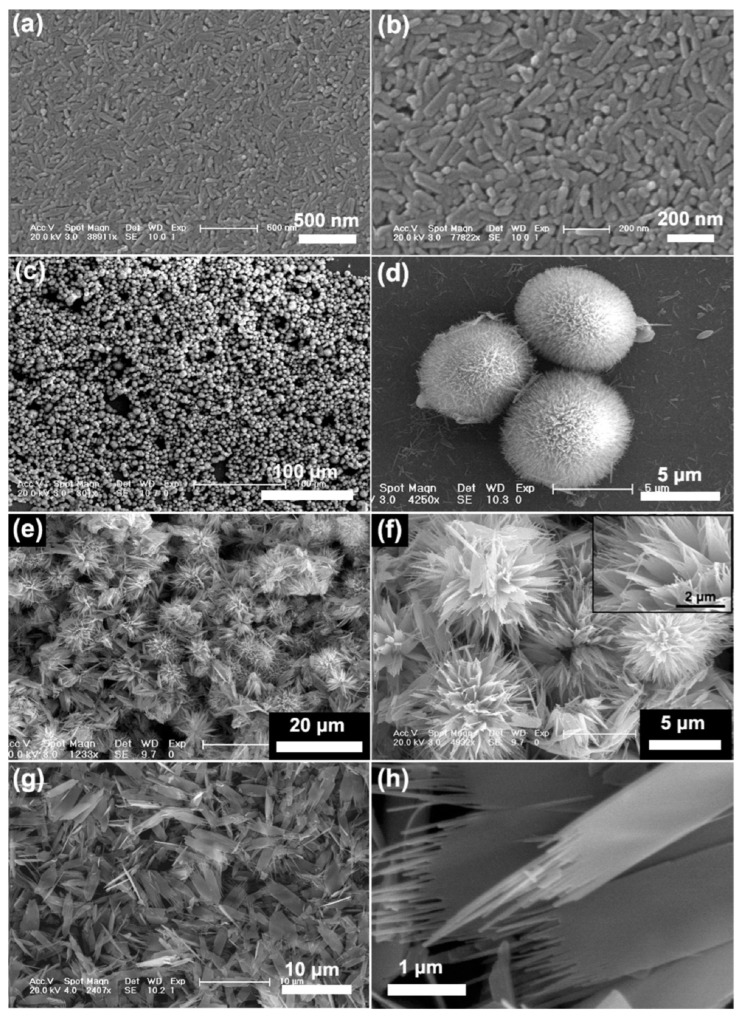
Nanorod (**a**,**b**), bur-like microsphere (**c**,**d**), microflower (**e**,**f**), and microsheet (**g**,**h**) morphologies that can be obtained from precipitation media having pH values of 7.0, 5.0. 4.5, and 4.0, respectively. Reprinted with permission from [19]. Copyright 2009 American Chemical Society.

Calcium phosphates (CaPs) and their composites with polymeric matrices appear as ideal mimicking materials for the regeneration of hard connective tissues (e.g., bones, teeth, and cartilages) due to intrinsic advantages that affect the mineralization process [13]. HAp is a naturally occurring CaP that is defined by the formula Ca_10_(PO_4_)_6_(OH)_2_ and that has an especial biological relevance. HAp clearly favors osteoinduction, osteoconduction, and osteointegration, which refer to the capacity of undifferentiated cells to render osteoblasts and osteocytes, the capacity to favor cell growth and the capacity to facilitate the contact between the living tissue and the implant, respectively [14]. Development of an appropriate material for tissue regeneration is complex and requires the appropriate combination of osteogenic cells, a biocompatible/biodegradable polymeric matrix with architecture and composition able to mimic the natural ECM and, finally, a vascularization to provide the transport of nutrients and metabolic products as well as bioactive agents [4,15]. HAp appears also an ideal compound for drug delivery applications since its nanoparticles (NPs) can be dissolved in acidic environments (e.g., lysosome vesicles of normal cells and extracellular media of cancer cells). Active compounds can be encapsulated into NPs during preparation (synthesis) or adsorbed on the surface, providing slow and fast releases, respectively. In any case, control of morphology (from needle to spherical shapes) is basic to tune the therapeutic behavior [16]. Synthetic HAp NPs can be prepared by precipitation methods according to simple processes, which also allow the control of morphology and size by small modifications of the experimental procedure [17,18]. Figure 5 displays, for example, the high variety of morphologies that can be achieved by only changing the pH of the precipitation solution. Rapid mixing of precursor solutions leads to the precipitation of amorphous calcium phosphate (ACP), which can subsequently be converted into the crystalline HAp form by a hydrothermal treatment [19,20], although other methodologies have also been successfully developed (e.g., self-assembly, spray drying, double emulsion, and sol–gel) [17,21,22,23].

Biomedical benefits derived from the implantation of materials incorporating HAp are strongly dependent on the ability to form interactions with the surrounding cells [24]. Therefore, surface treatments are habitual and also conside their effect on the adsorption and delivery of active pharmacological agents. Control of surface roughness and charge are primordial factors [25] that can be achieved by the incorporation of charged small compounds (e.g., citric acid [26]) and ions, such as Cu^2+^ and Ag^+^ which, in addition, can provide a bactericide effect [27]. Grafting of polymers on the HAp surface can also provide specific advantages such as the improvement of osteoconductivity [28], osteoblast differentiation [29], compatibility with the polymeric matrix [30], and control of cell differentiation by electric stimulation [31].

The use of HAp for drug delivery applications mainly concerns the encapsulation of bone morphogenetic proteins [32,33], fibroblast growth factors [33,34,35], alendronate as an antiosteoporosis agent [36], antibiotics (e.g., minocycline, doxycycline, dexamethasone, gentamicin, and erythromycin) [37,38,39,40], and vitamins [41]. Encapsulation of chloramphenicol (CAM) has recently taken relevancy since. In addition to being a wide-spectrum antibiotic, it can be used in cancer therapy due to the proven capacity of CAM to induce the mitochondrial dysfunction of cancer cells [42,43]. HAp NPs loaded with streptomycin antibiotics have recently been revealed to be appropriate against bacterial infections and appear also promising for the treatment of cancer cells [44]. 

**Figure 6:**
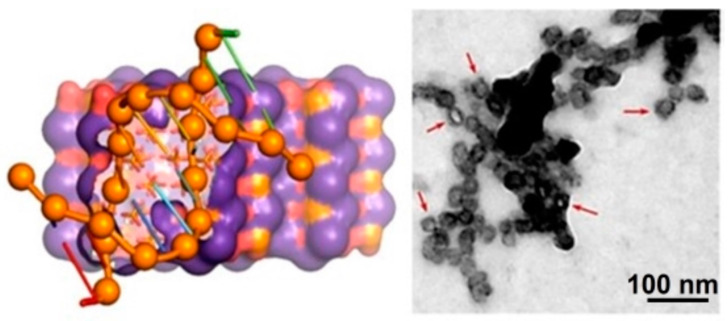
Scheme showing the incorporation of DNA inside the HAp structure (**left**) and TEM micrograph showing nanoparticles (red arrows) that incorporate DNA (**right**). Reproduced with permission from [48,49].

HAp NPs appear also promising as a non-viral gene vector due to their practically null immunogenicity and their availability to load transgenes with highly different sizes. HAp can also establish good interactions with DNA (basically through the interactions of calcium ions with the phosphate groups of DNA). Therefore, HAp becomes an ideal adsorbing and encapsulation agent [45,46]. Furthermore, HAp NPs seem to provide good protection for DNA (even against the Dnase digestion [47]) and seem able to incorporate DNA with a minimum distortion of its crystalline structure (Figure 6) [48].

## 3. Multifunctional Scaffolds

**Figure 7:**
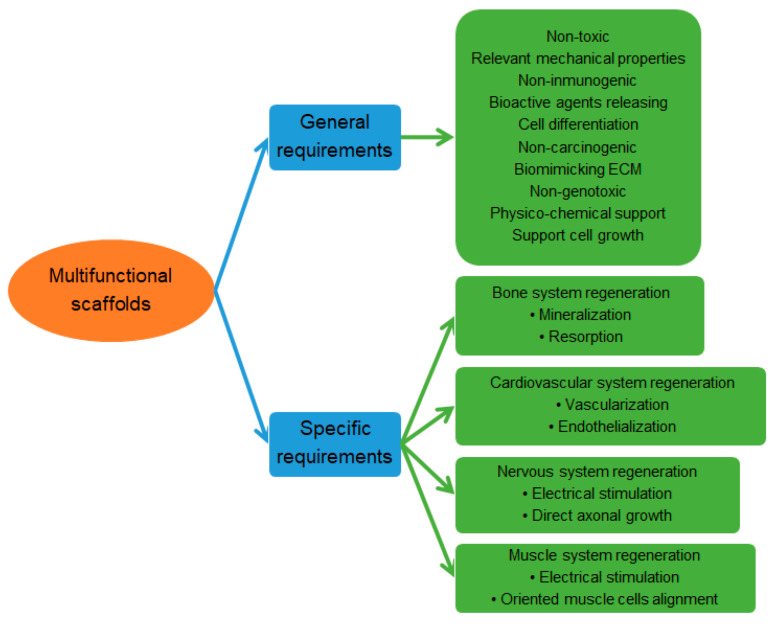
Scheme indicating the main requirements of bioscaffolds and their potential interest for different areas of tissue engineering. Adapted from [50].

The development of multifunctional bioscaffolds is, nowadays, one of the main concerns due to their implications on different areas of tissue engineering (e.g., cardiovascular, nervous, muscle, and bone tissues) through their capacity to deliver different bioactive molecules and to act as appropriate physicochemical support to a great variety of cells (Figure 7) [50]. 

**Figure 8:**
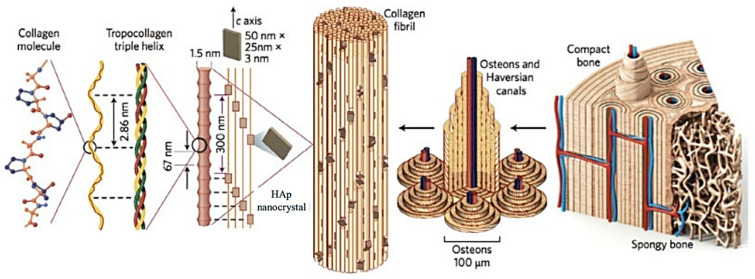
Scheme showing the hierarchical structure of bone with collagen and HAp as main components. Reproduced with permission from [55].

Bone tissue engineering (BTE) is one of the clearest examples of interest to develop 3D multifunctional scaffolds. These can prevent bone infections through local drug delivery and kill cancer cells located on bone defects (e.g., through hyperthermia or photothermal treatments), alongside the regeneration of the tissue [51,52,53,54]. Bone is a composite formed by organic components (mainly collagen I) and inorganic (mainly HAp) components. HAp is deposited in the collagen fibrils (the crystallographic *c*-axis being aligned with the axis of the fibril) and rendered osteons as a basic unit of bone (Figure 8) [55]. Bone has two macroscopic tissues: the cortical bone with low porosity and high mechanical properties, and the trabecular bone with a high porosity that allows contact with blood cells [56]. Techniques, such as 3D printing and electrospinning, appear nowadays ideal to reproduce the complex shape of a bone defect due to the precise control over microstructure and their great reproducibility. 

The success of tissue engineering strategies depends on an appropriate combination of biomaterials, (with or without surface functionalization), growth factors, and other active agents to stimulate the regeneration of the tissue and the neovessel formation (angiogenesis) after implantation [57]. 

Concerning preparation techniques, an excellent review has been performed by Bigham et al. [54]. There, both the traditional techniques (e.g., polymer sponge, leaching, freeze-drying, or foaming) and the procedures that allow a high control over microstructure are discussed (e.g., 3D printing and electrospinning). The role of stimuli to activate the multifunctionalized scaffolds to direct cellular behaviours is discussed in detail by Tai et al. [58]. The synthesis of architecturally controlled polymers seems a new great avenue for the development of scaffolds for tissue engineering. “Click” chemistry appears as an opportunity to develop bioactive materials (mainly based on proteins and peptides). Basically, “click” chemistry refers to a group of reactions that can be employed in a wide scope and have particular characteristics such as high yield, generation of inoffensive by-products, stereospecificity, and modularity [59]. For example, copper (I)-catalyzed alkyne-azide cycloaddition (CuAAC), strain-promoted alkyne-azide cycloaddition (SPAAC), thiol-X, Diels-Alder (DA), and oxime ligation methods have been discussed in detail, as well as their application in polymeric scaffold fabrication, has been reviewed by Zou et al. [60].

Three-dimensional printing technologies (not considered in the present review) represent, nowadays, one of the more powerful processes to produce functional scaffolds. Methods such as fused deposition modelling, stereolithography, selective laser sintering, and bioprinting have been discussed with a focus on tissue engineering [61].

### 3.1. Biopolymers and Synthetic Polymers

Type I collagen (Col) is the main organic constituent of the dermis, tendons, ligaments, dentin, and bone. COL1A1 and COL1A2 genes give rise to polymeric chains that subsequently are combined in the triple-stranded pro-collagen molecules. These are finally arranged forming compact, long, and thin fibers (diameters in the 100–200 nm range) that cross-link via lysine residues around cells [62,63]. Col for composite preparations is usually obtained from pig skin, bovine, or horse tendons. Col/HAp composites are mainly prepared according to two methodologies [64,65]. (a) The addition of HAp NPs to a Col suspension and subsequent lyophilization; and (b) the immersion of a porous Col scaffold into a suspension of HAp NPs and, subsequently, lyophilization. Multiple works demonstrated the ability of Col/HAp nanocomposites to stimulate the formation of new bone tissue [66]. Furthermore, these nanocomposites seem to act as a source of calcium cations that are incorporated into the regenerated tissue [67]. 

Interestingly, it has been observed that differentiation of osteoblasts into osteoclasts can be performed in Col/HAp membranes without the addition of other factors [68], a feature that contrasts with the observation carried out using only HAp. Probably, Col/HAp composites display exceptional properties (e.g., flexibility, high strength, biocompatibility bioactivity osteoconductivity, and bioresorbability [63]). Gelatin (Gel) derives from the degradation of the triple helix of Col that leads to single molecules. Applications are usually limited due to poor mechanical properties. Nevertheless, hydrogels exhibit biocompatibility and appear useful as good vehicles for cell transplantation after cross-linking with non-toxic enzymes [69]. Hyaluronic acid (HylA) is a linear and hydrophilic polymer constituted by N-acetyl glucosamine and glucuronic acid units. HylA has a high interest in bone regeneration due to its high elasticity, biocompatibility, degradability, osteoconductivity, and cell signalling function that enhances cell proliferation and differentiation [70,71]. HylA has also extended clinical applications as an injectable hydrogel that can subsequently be solidified using, for example, glutaraldehyde as a crosslinking agent [72]. Composite gels constituted by HylA copolymers and HAp have been developed for enhanced bone tissue regeneration [73]. Pyrogallol-conjugated HylA polymers appear ideal to obtain adhesive hydrogels containing HAp taking profit of the chelating properties of galloyl ligands [74]. HylA/HAp hydrogels also have promising applications as inks for bioprinting [75].

Chitosan (CS) is a linear copolymer of glucosamine and N-acetylglucosamine that is obtained from partial deacetylation under alkaline conditions of natural chitin [76]. Amino chitosan groups can interact with the calcium ions of HAp and act as nucleation sites for the growth of the inorganic component [77]. The first systems involving HAp and CS were applied for bone-filling cement [78]. The effectiveness of CS/HAp formulations has been demonstrated for the enhancement of in vivo bone tissue regeneration, delivery of stem cells, growth factors and bioactive drugs, and coatings to facilitate osseointegration [79,80,81,82]. Alginate (Alg) is a linear biopolymer composed of irregular blocks of *β*-D-mannuronate *α*-L-guluronnate residues that are mainly obtained from algae [83]. This biopolymer easily forms hydrogels through interactions with sodium and calcium ions, which have high applications for bone regeneration, drug delivery, and wound healing [84]. The addition of phosphate precursors to the calcium-Alg complex favours the HAp nucleation and leads to biocomposites with enhanced mechanical properties (e.g., hardness) and decreased porosity [85]. Alg/HAp composites seem appropriated for small-sized tissue defects, although the direct application for large bone defects needs to be improved [79].

Cellulose (Cel), the most abundant natural polymer on the earth and is composed of D-glucose units linked through glycosidic bonds. The linear syndiotactic chains can establish strong intermolecular hydrogen bonds giving rise to highly crystalline and insoluble materials. Interest in Cel and its derivatives is a consequence of their low cost, excellent mechanical properties, high porosity, biodegradability, and biocompatibility [86]. Bacterial nanocellulose or microbial cellulose has been proposed as an ideal nanomaterial for tissue engineering applications due to its wound-healing effect, fast tissue regeneration, and low inflammatory response [87,88]. Nanocellulose-based scaffolds functionalized with Col have shown excellent adhesion and proliferation of osteoblasts together with a high alkaline phosphatase expression and, consequently, are promising materials for tissue engineering [89]. HAp/bacterial Cel (BC) scaffolds have been considered an interesting biomimetic approach for bone-healing applications [90]. 

HAp NPs have similarly been combined with synthetic polymers in order to improve the mechanical properties of the scaffolds while maintaining the bioactive properties of the calcium phosphate. These NPs are normally combined with FDA-approved polymers. This is the case of polylactide (PLA) is currently the second most consumed bioplastic in the world due to its wide commodity and specialty applications. The polymer can be produced from renewable resources such as starch, corn, and sugarcane and is usually synthesized by the ring-opening polymerization of cyclic lactide dimers in the presence of metal catalysts. The stereoregular PLLA polymer (derived from L-lactide) is characterized by a glass transition temperature of 60–65 °C, a melting point of 175 °C, a crystallinity around 35%, and elastic modulus in the 2.5–16 GPa range [91]. These properties can be modified by the occurrence of racemization reactions, the synthesis from meso L-,D-lactide, or the random copolymerization of L-lactide and D-lactide monomer mixtures. Copolymers of lactide and glycolide (PLGA) have also a high relevance for biomedical applications due to their tuneable properties (e.g., degradation rate and degree of crystallinity) when the composition is varied [92].

**Figure 9:**
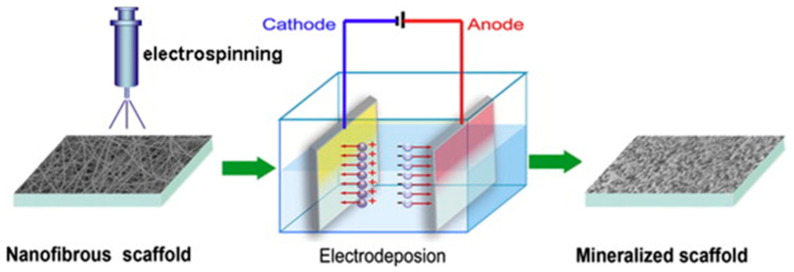
Experimental setup to obtain mineralized nanofibers by combining electrospinning and electrodeposition methods. The blue inset shows an SEM micrograph of a mineralized PLLA matrix prepared by electrodeposition at 3 V and 60 °C for 60 min. Reproduced with permission from [100].

PLA/HAp composites are able to modulate the cell environment in the function of the final structure of the system (coating, scaffold, fiber matrix, and hydrogel), allowing cell colonization and preserving mechanical properties until the regeneration of the tissue. The performance of PLA/HAp systems can be improved considering the capacity of both components to entrap molecules with biological activity. PLA/HAp systems can be easily prepared according to different processes/technologies, which can be specific. Examples include polymerization using HAp as an initiator, grafting of HAp on PLA oligomers, or more general processes such as electrodeposition, phase separation, and electrospinning. For example, an organic bone (i.e., HAp derived from mammalian bone) has been employed to induce a surface-initiated polymerization of lactide that led to composites with highly similar properties to bone tissues [93,94]. HAp NPs grafted with PLA can be obtained by direct polymerization of lactide onto the surface of NPs. Systems have a high potential to be used as bone fixation materials due to their good interfacial compatibility [95]. Grafting onto HAp surfaces can also be performed through ionic interactions between carboxylic terminal groups of PLA and Ca^2+^ ions of HAp and, therefore, the use of catalysts and coupling agents can be avoided [96,97]. Phase separation techniques have successfully been applied to mimic the nano-sized features of natural bone and obtain PLA/HAp materials with good mechanical properties and protein absorption capacity [98,99]. Applications of electrospinning will be extensively developed in the next sections, but it is interesting to point out the potential of electrodeposition of HAp over fiber-covered electrodes (Figure 9). The deposition was enhanced by electrochemical reactions and a local pH increase that led to a super-saturation of calcium phosphate and a fast formation of a HAp layer. A lower formation rate was characteristic after a conventional immersion on simulated body fluids (i.e., 12 days instead of 60 h) [100]. 

Poly(lactic-co-glycolic acid) PLGA/HAp composites have excellent in vitro properties due to the effects of nanoHAp (e.g., improvement of mechanical properties, an increase in water absorption, cell adhesion and proliferation, and high alkaline phosphatase activity) [101].

Polycaprolactone (PCL) and polyvinylalcohol (PVA) are two other polymers that have been widely applied in biomedical applications. The first one provides excellent mechanical properties but is highly hydrophobic and lacks cell recognition sites to favour cell attachment [102]. By contrast, the second one is hydrophilic and displays a high affinity for cell adhesion [103].

Polyhydroxyalkanoates (PHAs) are a family of biobased polyesters that can be obtained by bacterial fermentation [104]. Poly(3-hydroxybutyrate) (PHB), the shortest chain length member of PHAs, has received special attention due to its natural origin, and good performance that is combined with non-toxicity, biodegradability, and biocompatibility [105]. Despite the monomer having a chiral center, a single polymer configuration (enantiomer) is obtained by the biochemical process, a feature that leads to some limitations, such as high crystallinity and rigidity. Therefore, copolymers incorporating small percentages of other alkanoate units (e.g., poly(3-hydroxybutyrate-*co*-3-hydroxyvalerate) (PHBV)) are also considered to control the chain stiffness and increase both degradability and resorbability as a consequence of the reduction in the degree of crystallinity [106]. The main product of degradation, 3-hydroxybutyric acid, is beneficial for tissue engineering applications since it increases the calcium influx in cells [107]. Additional challenges of P3HB correspond to its relatively high cost of production, its lack of bioactivity [108,109], its hydrophobic character, and the difficulty to be processed due to the closeness between thermal degradation temperature and melting temperature (i.e., around 180 °C) [110]. Efforts to address these issues are nowadays justified considering the high potential of PHAs for biomedical applications as revealed by different research works concerning bone tissue engineering. PHB biomaterials show a piezoelectric character that favours in vivo bone growth [111]. Therefore, composites constituted by P3HB-based materials and HAp as a bioactive ceramic may provide an appropriate combination of mechanical and chemical properties [112]. 

Incorporation of HAp into P3HB composites has been proven effective to stimulate cell proliferation and to increase the growth and differentiation toward the osteoblast’s phenotype [113,114]. Different patches for the enhancement of bone regeneration have recently been developed [115,116,117,118], although no clinical trials have been performed since P3HB is not yet approved by the corresponding agencies (e.g., the Food and Drug Administration). These PHA/HAp composites led to a favourable bone tissue adaptation response without any chronic inflammatory effect in a 12-month evaluation period [119]. Bone quickly developed around the implant surface without being detected any in vivo structural breakage. In vitro studies using a simulated body fluid (SBF) showed the rapid development of bonelike apatite over a P3HB/HAp composite [120]. Bioactivity and, in general, mechanical properties can be modulated by modifying the amount of HAp. Composites from both P3HB and 3-hydroxybutyrate/3-hydroxyvalerate copolymers could achieve a compression modulus as high as 62 MPa (i.e., similar to human bone) and, therefore, seem adequate for the fixation of fractures [121].

Conductive polymers (e.g., polyaniline, polypyrrole, and polythiophene) have a relevant role in tissue regeneration (including bone) by allowing cells cultured on them to be stimulated by electrical signals. Usually, these polymers are blended with biodegradable polymers to improve mechanical properties and processability [122]. 

### 3.2. Surface Functionalization

The surface of Hap particles has usually been modified since it is a key factor that determines the capability to form appropriate interactions with surrounding cells [24]. In addition, protein adsorption and in general interactions with drugs and polymers depend obviously on the functionality and conformation of involved compounds, but also on the roughness, pore size, charge, and growth face of HAp NPs [25,123]. Calcium and phosphate ionic sites in the HAp surface can interact with COO^−^ and NH^3+^ groups of bone morphogenetic proteins, peptide growth factors (e.g., arginine-glycine-aspartate), and ionizable groups of active compounds (e.g., antibiotics). Furthermore, interactions able to improve compatibility can be established with polymer matrices having ionizable groups. Surface modification may consist of simple physical adsorption, but also a chemical immobilization such as the establishment of covalent links, ionic bombardment, or acid-base treatments. Precipitation processes can be used to immobilize amino acids and other compounds (e.g., mercapto succinic and citric acids) of distinct nature that lead to ideal acid or basic surfaces for the subsequent adsorption of negatively and positively charged compounds (e.g., the positively charged lysozyme can be easily adsorbed after treatment with aspartic acid, whereas bovine serum albumin requires treatment with arginine) [124].

Inorganic phosphate derivatives displaying biological functions can easily be incorporated onto HAp surfaces. Thus, the adsorption of bisphosphonates (BPs) is highly interesting due to their antiresorptive function, their capability to regulate calcium metabolism, and their ability to bind proteins (e.g., myoglobin and lysozyme) [125,126,127]. Pyrophosphoric acid is also an interesting surface modifier since has a great affinity towards basic proteins [128]. 

HAp lacks efficient protection from the immune system and, consequently, implants based on HAp have a relatively high risk of infection (i.e., implant surgery may represent around 50% of hospital-acquired infections [129]). Complications can lead to implant failure and even worse situations, such as amputation [130]. Therefore, the material should be integrated into the surrounding tissues before the formation of biofilms with high resistance to typical antibacterial agents (e.g., antibiotics). 

A good strategy is the incorporation of functional ions (i.e., Ag^+^, Zn^2+^, Cu^2+^, and SeO_3_^2−^) onto HAp by coprecipitation or by immersion (i.e., bulk or surface load). Basically, these doping agents can inhibit bacterial growth by binding thiol groups of enzymes, increase the production of reactive oxygen species (ROS), decrease the uptake of phosphate, or lead to DNA structural changes [131]. 

**Figure 10:**
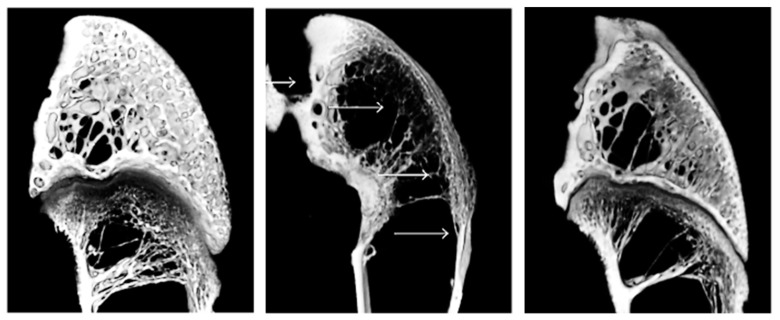
Micro-computed tomography graphs taken after 12 days of implantation of unloaded (**middle**) and VAN loaded (**right**) HAp/Col/calcium sulfate implants. White arrows indicated the tissue destruction in different places of the rabbit femoris. A normal femoris bone is shown on the (**left**). Reproduced with permission from [132].

HAp is an ideal compound for the load and release of antibacterial agents due to its great porosity. Usually, HAp is combined with natural and synthetic polymers (e.g., Alg, CS, Col, PVA, and ciclodextrines) due to its reduced mechanical response (i.e., high brittleness). Vancomycin (VAN) and gentamicin (GEN) are probably the most applied antibiotics for the treatment of bone tissue infections. Gram-positive bacteria such as *Staphylococcus aureus* and gram-negative bacteria, such as *Pseudomonas* and *Enterobacter* spp., are sensitive to VAN and GEM, respectively. Figure 10 shows, as an example, the effectiveness of a HAp/Col/calcium sulphate implant loaded with VAN for the reconstruction of a previously infected rabbit femoris bone [132].

The combination of effects caused by doping with antibacterial ions (mainly Ag^+^) and the incorporation of antibiotics (e.g., ciprofloxacin, tetracycline, and VAN) has also been revealed to be effective. Thus, prolonged antibacterial activity and increased efficiency have been reported in some cases [133]. 

The surface of PHAs has been modified by physical or chemical immobilization of Col in order to increase cell proliferation [134]. C^+^ ion implantation was also effective to increase biocompatibility with fibroblast cells [135].

### 3.3. Incorporation of Functional Compounds

Bone remodelling is a crucial process that avoids health problems, such as osteoporosis, and allows effective hard tissue regeneration. It is triggered by osteocytes, which detect bone defects (e.g., microcracks, mechanical strains, etc.), and involves two opposite processes: (a) bone resorption controlled by osteoclasts, and (b) bone formation controlled by osteoblasts. Thus, osteoblasts mineralize the cavities previously produced by osteoclasts [136] once the bone defects were detected by osteocytes.

Different natural compounds can be used as osteogenesis inducers. The main effects are the inhibition of bone resorption and the enhancement of bone formation and maturation [136]. Different flavonoids (polyphenols obtained from plants) prevent bone resorption processes while promoting osteoblastogenesis [137] (facilitating differentiation of mesenchymal cells (MSCs) into osteoblasts). The most representative examples are (a) epigallocatechin-3-gallate, obtained from the plant *Camellia sinensis*, which shows a great capacity to induce cell proliferation, osteogenesis, and mineralization [138], together with anti-inflammatory, antibacterial, and antioxidant functionalities [139,140]; (b) acemannan, obtained from plant *Aloe vera*, has a well-demonstrated capacity to increase both cell proliferation and bone healing rate, and lead to a fast bone and ligament regeneration [141,142]; (c) icariin, an active agent obtained from *Herba Epimediian*, which promotes osteoinductivity (e.g., Alg/HAp [143] and PLGA/β-calcium phosphate [144] systems); (d) curcumin, the main component of plant *Curcuma longa*, is a well-known agent for the treatment of different pathologies due to antibacterial, antioxidant, and healing properties [145] which enhance cell differentiation, proliferation, and migration and also has beneficial effects in the treatment of diabetes [146] and osteosarcoma patients [147]; and (e) revesratrol, a component of fruits such as grapes, berries, and nuts, which improves the blood supply to bones [148], facilitates the expression of endothelial growth factors and the osteogenic differentiation, has beneficial effects decreasing tumorigenicity against cancer cells [149], and shows a high potential for bone and cartilage regeneration [150,151]. 

Incorporation of growth factors (e.g., the bone morphogenetic protein BMP-2) into HAp NPs is also an effective way to induce osteogenesis of mesenchymal cells and to promote adequate vascularization [152]. The vascular endothelial growth factor (VEGF) plays an essential role in the regulation of angiogenesis and has, consequently, been used for tissue engineering applications. Surface immobilized growth factors may have advantages related to greater stability and prolonged function. Therefore, different studies have been focused on the immobilization of VEGF onto HAp NPs [153]. Results demonstrated a local regulation of the cell response and the improvement of adhesion and proliferation of endothelial progenitor cells involved in revascularization processes [154]. The acceleration of bone growth and the healing of defects can be possible through the incorporation of fibroblast growth factors [155]. Growth factor proteins are usually substituted by the active peptide sequences due to their high cost and preservation difficulties. Therefore, peptides with the core active sequence of BMP-2 (24 amino acids) were found adequate to mimic the properties of the complete protein [156]. The arginine-glycine-aspartate (RGD) peptide is the principal integrin-binding domain present within the extracellular matrix proteins. RGD has consequently been widely employed to functionalize HAp and promote the adhesion and survival of cells involved in the regeneration process (e.g., fibronectin, vitronectin, and fibrinogen). However, some cautions should be taken into account due to some inhibitory effects on bone formation [157]. Interestingly in some cases, the selected peptide sequence can render better results than the morphogenetic protein. Thus, a 15 amino acid sequence of an active region of BMP-7 gave rise to a better osteogenic activity than the complete protein [158]. 

## 4. Electrospinning

**Figure 11:**
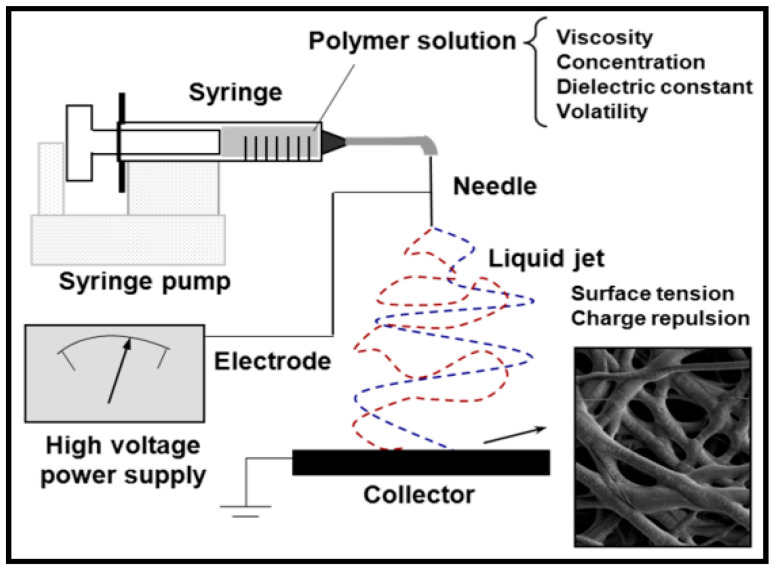
Schematic diagram showing the main parts of typical electrospinning equipment. Reproduced with permission from [170].

Electrospinning is a suitable technique to prepare easily fibers from a wide range of polymeric materials. These fibers have diameters that can vary from the micrometer to the nanometer scale [159,160,161,162,163,164,165,166,167,168,169]. The basic equipment requires a high-voltage source to charge the surface of a polymer solution droplet, a micro-dosing pump to continuously feed the solution to the end of a capillary tube (usually, a hypodermic needle with a blunt tip), and a grounded target (conductive collector) (Figure 11) [170].

Droplets are deformed into a Taylor cone when the electrostatic repulsion among the generated surface charges overcomes the surface tension. After that, a jet is ejected from the needle towards the collector. This jet splits into multiple filaments due to the radial charge repulsion and gives rise to solidified ultrathin fibers after the evaporation of the volatile solvent.

Multiple factors can be controlled in order to process the different polymeric systems and render fibers with a selected morphology. Thus, the geometry of the electrospinning equipment (e.g., horizontal or vertical plane collectors or rotating collector), solution properties (e.g., viscosity, dielectric constant, volatility, and polymer concentration), and operational parameters (e.g., the strength of the applied electrical field, deposition distance, flow rate, and temperature) should be carefully optimized [161,163,164,165,166]. Electrospinning favours molecular orientation due to the high elongation rate and cross-sectional area reduction in the formed fibers. The deposition of electrospun fibers leads to networks with interconnected pores that are interesting for different biomedical applications (e.g., drug delivery, wound dressings, and blood vessels). Extracellular matrices (ECMs) can be well mimicked by the electrospun fibers due to their similarity with Col. Therefore, tissue regeneration becomes one of the most important uses of electrospun scaffolds, especially considering the ability to control topography (e.g., fiber diameter, diameter distribution, fiber alignment, and porosity) [171,172,173,174] and to produce a surface functionalization. Furthermore, the capacity to encapsulate and render a local release of different agents (e.g., antioxidants, anti-inflammatory, bactericides, and growth factors) should be taken into account. 

Scaffolds prepared by conventional electrospinning are constituted of tightly packed layers of fibers, a feature that can be a serious limitation for tissue engineering. Porosity is mainly located at the surface of the scaffold and, consequently, the cell growth inside the material is seriously hindered. Efforts are nowadays focused to provide a real 3D structure while keeping interconnected pores and nanofibrous morphology [175,176]. Different methodologies have been reported to generate real 3D scaffolds by means of electrospinning: multilayered assembly [177], template-assisted electrospinning [178], incorporation of porogen agents [179], and post-treated systems (e.g, tubular scaffolds from rolling the initial flat layers) [180]. Furthermore, other approaches are being developed by combining electrospinning with 3D forming technologies [181,182]. Thus, multiscale scaffolds composed of micro and nanoscale structures have been developed [183]. These macrostructures have good interconnectivity, high porosity, and nanoscale features that allow for enhancing cell attachment. 

**Figure 12:**
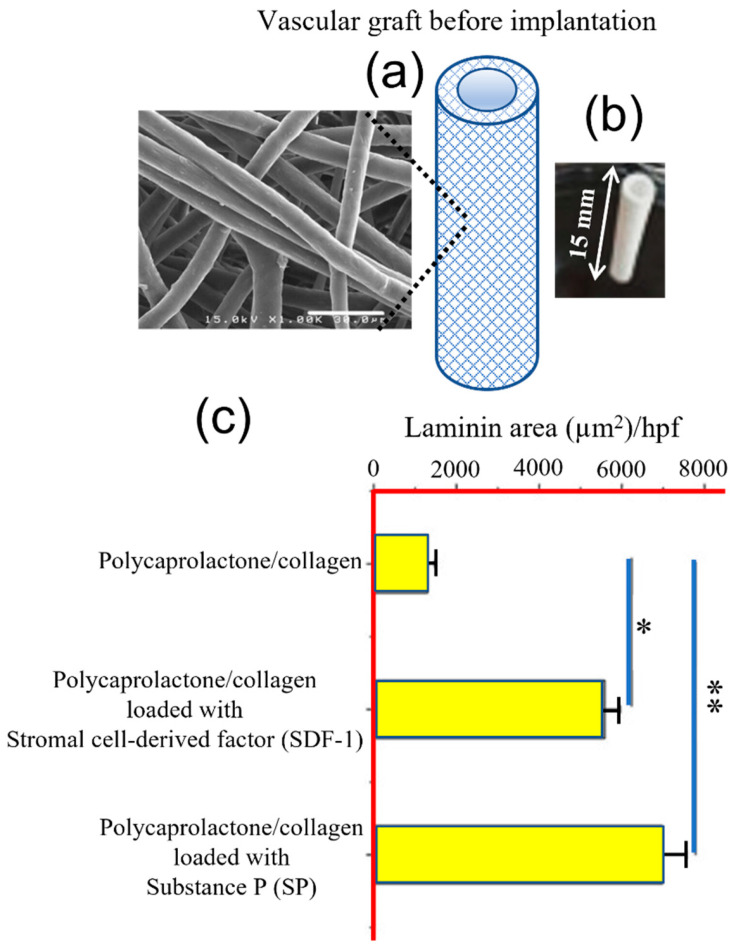
Vascular graft fabricated by electrospinning (**a**,**b**). Grafts loaded with SP and even SDF-1 had a significantly higher ability to recruit cells (evaluated through the laminin area) to the injury site than the control (PCL/Col) (**c**). Adapted from [185].

The application of electrospinning to develop tissue engineering scaffolds is extensive, noticeable in the capability to load different bioactive agents. Thus, conveniently loaded scaffolds can promote the formation of blood vessels during wound repair, as reviewed by Wu et al. [184]. Specifically, grafts fabricated by electrospinning and loaded with stromal cell-derived factor 1 (SDF1) or the specificity protein 1 (SP1) showed a clear enhancement of angiogenesis with respect to unloaded controls (Figure 12) [185].

Electrospun scaffolds functionalized with inorganic, organic, or bioactive agents appear ideal for tissue engineering applications since outstanding physicochemical and biological properties can be derived. An outlook concerning the development of such electrospun composite nanofibers has recently been reported [186].

Bioelectronic sensors have also been developed taking into account that electrospun fibers may display piezoelectric properties and provide an accurate measurement of static pressure on the skin [182,187]. Electrospun scaffolds can also be in situ deposited on wound surfaces for minimally invasive operations [182,188].

**Figure 13:**
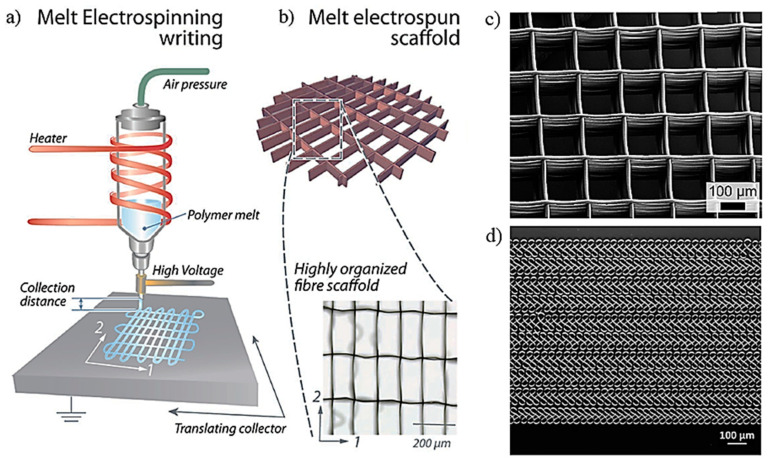
(**a**) Scheme of a MEW device showing the main parts: dispensing; electrical heating system; high-voltage source electrode; and computer-assisted collector plate. (**b**) Scheme of a melt electrospun fiber scaffold. (**c**,**d**) corresponds to micrographs of different types of melt electrowritten samples. Reproduced with permission from [194,195].

Electrospinning can also be carried out from a molten polymer instead of a polymer solution. Physical principles are similar but there are important differences that concern the high viscosity of the melt and the necessity to heat the polymer and then cool rapidly the electrified jet that is formed. This process, called melt electrospinning (MES), offers some advantages over the traditional solution electrospinning, such as the absence of toxic solvents and a higher possibility to generate 3D structures due to both the usually higher diameters of the generated fibers and the more foaming/spongy texture of the deposited scaffold. Nevertheless, there are limitations concerning thermal degradation and even great difficulty to control the final morphology [189,190,191,192,193]. Melt electrospinning writing (MEW) is a different configuration where the extruded filaments are deposited onto the collector according to determined program instructions that allow building ordered and predefined architectures (Figure 13). Basically, this 3D printing technology combines the principles of electrospinning and additive manufacturing [194,195].

### 4.1. Coaxial Electrospinning

**Figure 14:**
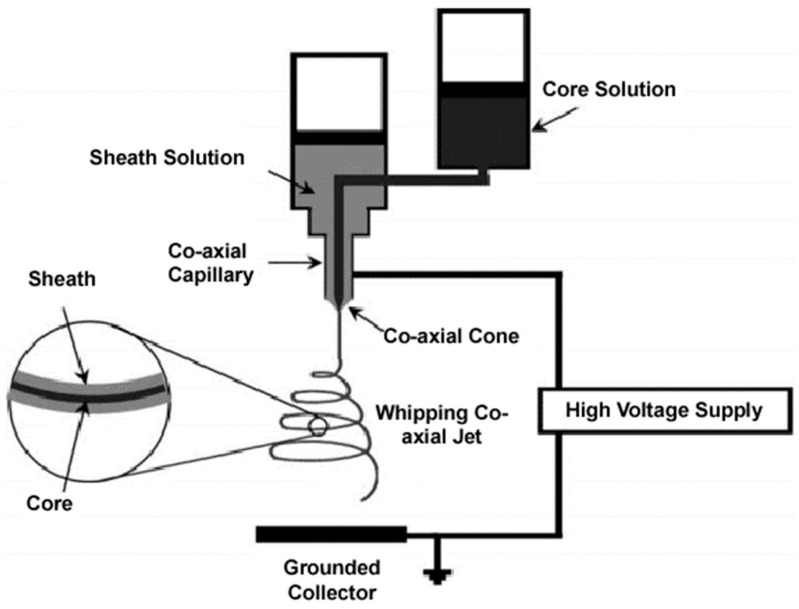
Scheme of coaxial electrospinning equipment and the derived core–shell fiber morphology. Reproduced with permission from [198].

Complex and diverse fiber morphologies can be obtained by means of coaxial electrospinning, a technique that was first introduced in the early 2000s [196]. Internal fiber structures can be varied and classified as hollow [197], core–shell [198], tube-in-tube [199], and multi-layered/multi-channeled [200]. Probably, the core–shell structure is the simplest and the most studied system. The basic electrospinning equipment only needs to be modified, in this case by the use of two feeding solutions and a coaxial capillary (Figure 14). The success of the process depends on geometric parameters such as the inner/outer nozzle diameter ratio, the separation distance between the two nozzles, and the length of the nozzles [201,202], as well as physicochemical and operational parameters. Thus, interfacial properties (e.g., miscibility and compatibility between solutions), flow rate, and viscosity ratios between the two polymeric solutions are fundamental. The viscosity of the shell solution should be high in order to overcome the interface surface tension and to render a stable structure, while the flow rate of this solution must be also high in order to stretch continuously the core solution continuously [203,204].

Different core–shell systems have been evaluated for biomedical applications. In this way, fibers with a CS shell and a PLA core have been produced, considering that the latter could favor the electrospinnability of the former [205]. In a similar way, nanofibers of the rubbery poly(glycerol sebacate) (PGS) could only be produced by means of co-electrospinning [206]. In this case, PLA was employed as a shell material able to constrain PGS and even facilitate its processing. In other cases, the new coaxial fibers have better mechanical properties than the individual components (e.g., the Gel/PCL core/shell system [207]). Similarly, the morphology and performance (adhesion and mechanical strength) of polycarbonate (PC) nanofibers can be improved when they are recovered by a polyurethane shell [208]. Coaxial fibers appear also ideal for sustained drug delivery since bioactive agents (e.g., antibiotics, antioxidants, and growth factors) can be loaded in a core that is protected by the outer shell layer that can minimize the initial burst effect [209,210,211,212].

Hollow fibers can be produced when the core is selectively dissolved or thermally decomposed [213,214]. Thus, CS, PLA, and PCL hollow fibers can be produced using water-soluble polymers, such as polyethylene glycol/polyethylene oxide (PEO), for the core [212,215,216]. Indeed, mineral oils have been used for the core as is the case of cellulose acetate (CA) hollow fibers [217]. The incorporation of functional compounds may allow us to obtain functionalized fibers at both inner and outer surfaces as a consequence of a simple diffusion process [199]. 

Complex fibers having a great capacity to tune final properties can be achieved by means of multicoaxial electrospinning. This allows us to obtain multilayered, multichanneled, and even tube-in-tube (using an intermediate layer able to be solubilized) nanofibers. Possibilities of this kind of architecture are noticeable since the properties of each individual layer can be varied (e.g., hydrophilic and conductive properties) giving materials with high biocompatibility, high mechanical properties, high functionality, and sustained drug release [199,218,219,220]. Nevertheless, the difficulty to control accurately the complex process is still limiting its applicability. The development of new needleless electrospinning processes may be an interesting approach to obtain these complex multi-layered structures [221,222].

### 4.2. Emulsion Electrospinning

**Figure 15:**
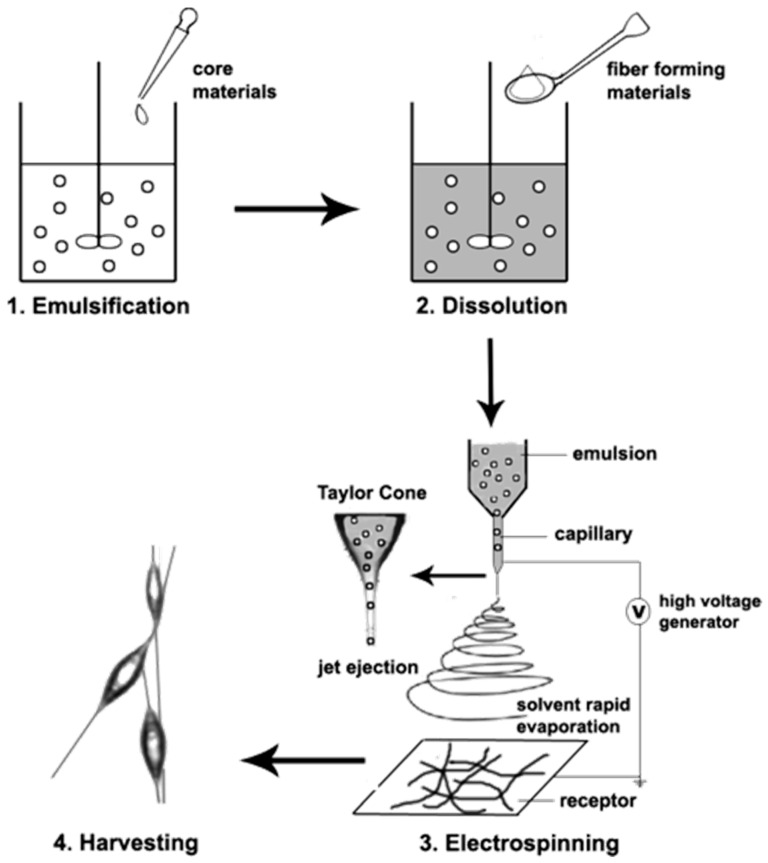
Scheme of a simple emulsion electrospinning process. Reproduced with permission from [223].

Nanofibers with a core–shell structure can also be obtained using a single nozzle if an emulsion of two solutions is properly processed. The main steps involved in this case are the emulsification step to form a water/oil emulsion, the dissolution of a fiber-forming polymer in the organic medium and, finally, the electrospinning of the emulsion (Figure 15) [223]. Basically, the continuous phase (shell) usually corresponds to a solution of a biodegradable polymer in an organic solvent, whereas an aqueous medium is usually employed to dissolve the active agent. Small or high molecular weight bioactive compounds will render single fiber or core–shell structures, respectively. Continuous evaporation of the organic phase during electrospinning increases the viscosity of this phase and favours the migration of aqueous phase droplets to the center of the fiber. In this way, an inner column is formed if the migrated phase contains a high molecular weight polymer. Emulsion electrospinning has the possibility to reduce the number of organic solvents as an additional advantage with respect to coaxial electrospinning [224,225]. The selection of an appropriate polymer for the shell is fundamental to obtaining a correct drug encapsulation. For example, PLA tends to form a pothole-like surface structure that led to an incomplete encapsulation of hydrophilic drugs. This problem may be solved using an amphiphilic block copolymer of PLA and PEG since a uniform shell morphology can be attained and, therefore, efficient incorporation of the selected drug in the core is derived [226]. 

**Figure 16:**
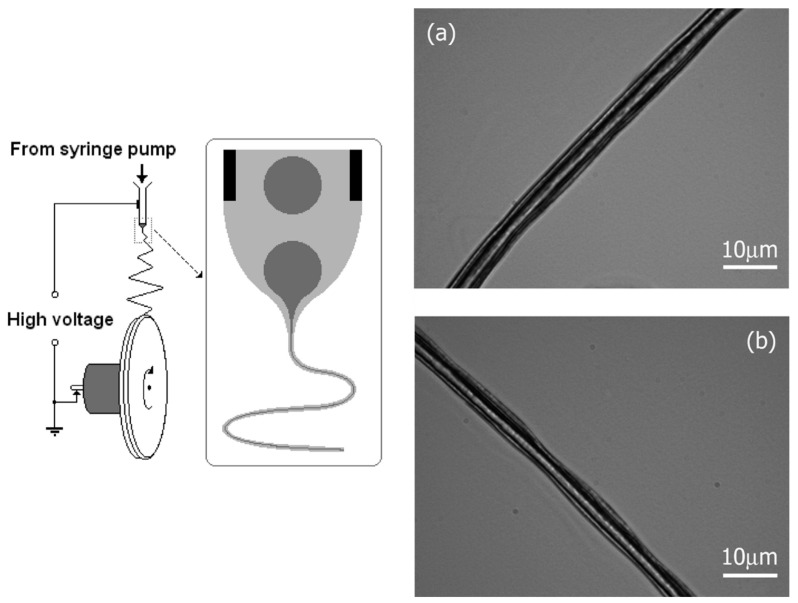
Optical micrograph showing the core–shell structure obtained from electrospinning the emulsion of PMMA/DMF in PAN/DMF (**right**). Note that initial droplets (dark gray) that are shown in the needle detail led to a continuous filament after electrospinning. In (**a**,**b**), optical images of the as-spun core–shell (PMMA-PAN) microfibers can be observed. The fiber in (**a**) has a relative more uniform inner/outer diameter than (**b**). Reproduced with permission from [227].

It should be pointed out that the use of two immiscible solvents is not a strict requirement, since it is only necessary that a phase separation could be spontaneously produced after mixing the two polymer solutions. For example, a single solution of polymethylmethacrylate (PMMA) and polyacrylonitrile (PAN) in dimethylformamide (DMF) leads, after blending, to phase separation and the formation of dispersed droplets of the minor component solution (e.g., PMMA/DMF) into the continuous PAN/DMF phase [227]. Droplets lead to a continuous inner fiber (Figure 16) due to both the high stretching of confined droplets in the co-electrospun jet and the relatively low flow rate that allows the charge relaxation of the polymer solution (i.e., traction becomes a consequence of viscous forces) [228]. 

A relation between the concentration of the emulsified compound and the relative diameter of the core has been indicated [229]. Furthermore, droplets with high initial diameters could not be properly stretched and thick fibers with discontinuous cores are obtained. In all cases, the volumetric ratio between the core and the shell cannot be higher than the corresponding weight ratio of the solid compounds in the formulation. On the contrary, the hydrophobic shell polymer will migrate towards the core as a consequence of the higher volatility of the organic solvent and the confinement derived from the faster solidification of the shell [229,230].

## 5. Recent Developments on Multifunctional Electrospun Scaffolds Incorporating HAp

Different scaffolds have been developed in the past year that aimed to incorporate HAp to improve the remodelling and repairing of the bone tissue. Some of these attempts are summarized in Table 1. 

Multifunctional layered scaffolds with potential applications for nasal cartilages and subchondral bone reconstruction have been prepared using PLA and Gel as biomaterials by combining 3D printing (e.g., fused deposition modelling) and electrospinning fabrication techniques [231]. Specifically, a Gel solution with and without a commercial osteogenon drug (ossein-HAp complex (OHC) also containing osteocalcin and type I Col) was electrospun over a 3D printed PLA scaffold. The surface of the scaffold has, therefore, the hydrogel characteristics highly recommended to mimic the natural and nanofibrous environment of cartilage. In addition, Gel provided integrin binding sites that favoured cell adhesion and offered some advantages with respect to Col (e.g., low immunogenicity and pathogen transmission problems [232]). New constructs were bioactive since both mineralization and cell adhesion was enhanced. 

Promising scaffolds for tissue engineering have been prepared by electrospinning mixtures of PLLA, Col, and HAp. The inorganic component was essential to improve the tensile properties of the scaffold [233], while the final structure could satisfactorily mimic the nanoscale structure of the ECM. 

The periosteum membrane is the outer layer of bones and consequently plays a crucial function in the transmission of molecular information between the bone and the surrounding muscles [234]. The membrane is essential for bone growth and regeneration, as it provides the osteogenic cells necessary to repair defects and excludes cells that prevent bone formation [235]. The structure of natural periosteum is complex since involves three different layers: the inner layer that favours osteogenicity and is constituted by osteoblasts and Col, the voluminous interlayer where undifferentiated and progenitor cells are stored and, finally, the outer layer that has the highest content on Col fibrils [236]. Different attends, which were mainly based on electrospinning processes, have been evaluated to obtain membranes able to mimic the periosteum. In this way, it has been designed/evaluated a trilayered system constituted by electrospun fibers of PCL (outer layer), a mixture of polyurethane and PCL (intermediate layer), and a mixture of polyurethane and nanoHAp (inner layer) [237]. Basically, the outer layer should prevent cell permeation and should have a very slow degradation rate, while the interlayer and the inner layers should be successively degraded. In addition, the inner layer should promote bone regeneration. Results demonstrated that suitable mechanical properties increasing from the inner to the outer layer and degradation rates increasing in the opposite direction were achieved. Furthermore, the system demonstrated appropriate multifunctionality and response for tissue regeneration. It is significant that the small porous structure and slow degradation rate prevented cell infiltration in the outer PCL layer, but the fast degradation of the other layers provided enough space for cell ingrowth. 

Complex multifunctional scaffolds were obtained by the self-polymerization of dopamine (DA) on the surface of PLLA/HAp electrospun fibers [238]. The adhesive membrane was subsequently coated with polypyrrole (PPy) via an electrochemical process that allowed the chelation and coordination of silver ions. The final PLLA/HAp/PDA/PPy/Ag composite showed long-term antibacterial properties, bioactivity, and osteoinductivity. 

Core–shell electrospun fibers having a shell of PLA/HAp and a core of PCL have been investigated for bone tissue engineering [239]. PCL was selected for its slower degradation rate, ductile character, and for providing mechanical stability to the fiber. The addition of HAp was interesting since its break products are basic and, consequently, can neutralize the acid medium generated by the degradation of both polyesters [240]. The two polymers could be solubilized using the same solvent mixture (i.e., chloroform/acetone) and, consequently, a good interface between both polymers was obtained. Interestingly, bioactivity was enhanced when the core flow rate was increased since Hap particles were forced to protrude out of the fiber surface. A sustained release of BMP-2 (96 h) was observed for these new scaffolds that also allowed MSC attachment and supported osteogenic differentiation. 

The advantages of coaxial over monoaxial electrospinning have been emphasized in the comparative study performed with the PCL/Gel/Poloxamer 188 scaffold that was designed for bone tissue applications [241]. Specifically, P-188 is a copolymer able to provide anti-thrombotic, anti-inflammatory, and cytoprotective activities to injured tissues [242]. Coaxial fibers were efficient for a dual release involving a hydrophilic protein (β-lactoglobulin) and a hydrophobic agent (vitamin K2). Furthermore, scaffolds showed good stability, no significant swelling, and enhanced Saos-2 cell viability. 

Composites of silk fibroin nanofibers with HAp have been proposed to mimic natural bone [243], demonstrating biocompatibility and good mechanical performance. Nevertheless, progress is still necessary to obtain scaffolds with appropriate bone-like architecture. In this sense, the selection of the type of silk seems to be a crucial factor that determines the quality of the final scaffold [243]. Specifically, tussah silk fibroin (TSF) contains the Arg-Gly-Asp motif, which plays a significant role as a biological recognition signal [244] and promotes fibroblast cell adhesion [245]. Natural fibrils in natural bone have recently been mimicked by means of coaxial electrospinning [246]. The nanostructured fibers consisted of a core of a HAp/TSF composite and a shell of TS. Results indicated a good mechanical performance and improved cell adhesion, proliferation, and bone formation capacity tan pure silk. Nevertheless, TSF has some intrinsic problems derived from the crystallinity decrease during the regeneration process and, consequently, the loss of mechanical properties. In order to solve this problem, blends of TSF with other polymers, such as cellulose acetate (CA), have been proposed [247] In fact, CA has shown appropriate properties to also be employed for bone implants [248]. A bone tissue engineering scaffold has recently been fabricated by Tao et al. [249] through coaxial electrospinning. The core was constituted by CA, while the shell consisted of a mixture of TSF and PEO loaded with HAp and BMP-2. Scaffolds had micro/submicro structures and better mechanical properties than materials produced with silk as a single component. BMF-2 was quickly released at the beginning but then a sustained delivery as long as three weeks was observed. HAp and BMF-2 played a fundamental role in promoting osteogenic differentiation. In vivo studies demonstrated enhanced bone regeneration after 12 weeks of implantation. 

Citrate-stabilized gold-nanoparticles (GNPs) have been encapsulated into new coaxial electrospun scaffolds constituted by polyvinylpyrrolidone (shell) and ethylcellulose (core) [250]. Appropriate incorporation of GNP in the cell rendered promising in vitro and in vivo results concerning bone tissue regeneration. Porosity, mechanical performance, biocompatibility, and osteogenic activity were clearly enhanced after GNP incorporation. Results corroborate the positive effect of GNPs on alkaline phosphatase activity [251] and the previous in vivo findings about the acceleration of bone regeneration [252]. 

A core–shell fibrous membrane with a great capacity to immobilize heparin has been prepared by coaxial electrospinning [253]. The shell of the fiber was constituted by cationized Gel that was subsequently crosslinked by exposure to glutaraldehyde vapors, while the core consisted of PCL in order to improve the mechanical properties of the hydrogel. The VEGF endothelial growth factor could be effectively impregnated into the fibers through specific interactions with the immobilized heparin. These interactions slowed down the release of the growth factor in such a way that a sustained release was achieved for more than 15 days. New materials were highly interesting due to the presence of multiple angioactive molecules (i.e., Gel, heparin, and VEGF) and the hemocompatible surface. 

A complex electrospun scaffold consisting of coaxial nanofibers having a core of PVA incorporating oregano extract and mesoporous silica nanoparticles (PVA-OE-MSNPs) and a shell constituted by a PCL/Col blend incorporating HAp has recently been evaluated for hard tissue engineering [254]. In addition to the benefits provided by Col and HAp, it should be noted the characteristics of the hydrophilic core brought by mesoporous silica nanoparticles since they can promote osteoconduction [255] and control the release of the selected extract. This extract has high terpene and phenolic content and, consequently, provided antioxidant, anti-inflammatory, and antibacterial properties together with a positive impact on bone metabolism.

Guided tissue regeneration (GTR) is employed to solve problems caused by the fast fibroblast growth and the derived interference when these cells fill the defect sites of bone, preventing the growth and healing of new bone [256]. GTR makes usually use of appropriate membranes that cover the area between bone and soft tissue. These membranes can be fully biodegradable or, on the contrary, non-biodegradable, being in this case required a second surgery for their removal. The biodegradable membranes should keep their function at least for 2 months to prevent fibroblast growth into defects and should have multifunctional properties, such as antibacterial characteristics and the capacity to induce bone regeneration. Polymers such as PLA, PLGA, and PCL have been proposed and techniques, such as electrospinning, appear ideal due to their compact structure and similarity with the ECM [257,258]. Nevertheless, the above-indicated requirements are difficult to be accomplished with a single component, as they are necessary to work on more complex systems. Furthermore, synthetic polymers have intrinsic problems such as high hydrophobicity, low stiffness, relatively low bioactivity, and certain toxicity of degradation products, while natural polymers may present a much too high degradation rate [259]. Fewer efforts have been focused on the development of systems that combine barrier properties, suitable drug release, and enhanced bone growth induction. Tang et al. [260] have recently developed multifunctional nanofiber membranes by coaxial electrospinning. These membranes consisted of a core of PLGA/HAp and a shell of Col/amoxicillin, as they were determined to be promising barrier properties for this system. Basically, the core was able to block and promote fibroblast and bone growth, respectively, while the shell favoured wound healing through the release of the selected drug. Barrier properties were demonstrated since fibroblasts grew well on the side of the membrane where they were cultured, while no fibroblasts were observed on the opposite side. 

Fibrous scaffolds having a PCL core and a shell of PVA loaded with HAp NPs have recently been produced using a new spinning technique [261]. In this case, the corresponding polymer solutions were extruded trough concentric nozzles under the action of rotation and pressure [262]. The lack of limitations on the selection of solvents appears the main advantage over conventional electrospinning. Furthermore, the electric field can be avoided, the cost can be reduced, production capacity can be increased and, overall, the surface of the material can be easily functionalized. 

Microparticles constituted by a core of CS/HAp and a shell of zein (a prolamine plant protein) loaded with simvastatin (SIM) were prepared by coaxial electrospraying [263]. These particles with diameters in the range of 1 μm were fully composed of natural materials and provided a long-term sustained release activity as a consequence of their double-layer structure. Furthermore, cell proliferation and osteogenic properties were enhanced.

**Table 1:** Multifunctional electrospun scaffolds.

Multifunctional Composite	Applications/Effects	Ref.
PLA, osteogenon^TM^ (ossein, HAp), osteocalcin, Col I	Hydrogel for cartilage reparation. The mineralization and cell adhesion enhanced.	[231]
PLLA, Col, HAp	Reparation of bone periosteum. Improve tensile properties and mimic the nanoscale structure of the extracellular matrix.	[233]
PCL, polyurethane, nanoHAp	Promotion of bone regeneration. Controlled degradation and cell ingrowth.	[237]
PLLA, HAp, dopamine, PPy, Ag	This showed long-term antibacterial, bioactivity, and osteoconductivity properties.	[238]
PLA, HAp, PCL, BMP-2	Bone tissue engineering by slower degradation, acid neutralization, and the enhancement of the mesenquimal cell attachment and osteogenic differentiation.	[239]
PCL, Gel, poloxamer 188, β-lactoglobulin, vitamin K12	The scaffold provides anti-thrombotic, anti-inflammatory, and cytoprotective activities	[241]
Silk fibroin, HAp,	Biocompatibility and good mechanical performance. Bone-like architecture.	[243]
Silk fibroin, CA	Employed for bone implants.	[247]
CA, silk fibroin, PEO, Hap, BMF-2	Improve mechanical properties. Promotion of osteogenic differentiation.	[249]
PVP, ethylcellulose, Au NPs	Bone tissue regeneration. The porosity, mechanical performance, biocompatibility, and osteogenic activity was enhanced.	[250]
PCL, Gel, heparin, VEGF	Hydrogel with angioactive molecules and hemocompatible surface.	[253]
PVA, PCL, oregano oil, silica, HAp	Provide antioxidant, anti-inflammatory, and antibacterial properties.	[254]
PLGA, HAp, Col, amoxicillin	Promote fibroblast, bone growth, and wound healing.	[260]

Titania (TiO_2_) is one of the most employed inorganic biomaterials due to its excellent biocompatibility [264]. Titanium composites with calcium phosphate compounds have also a great interest in joint prostheses due to their appropriate compressive strength and biocompatibility [265]. Titanium can be electrospun, generally in combination with selected polymers, to form ultrathin fibers. Subsequent calcination processes may lead to hollow nanofibers which have a well-demonstrated interest as implant materials thanks to their high mass transfer capability (i.e., transportation of biomacromolecules) and a reasonable surface porosity that enhances the interaction with the surrounding tissues [266]. Highly porous, hollow TiO_2_ nanofibers (NFs) were fabricated through coaxial electrospinning using a solution of a titanium precursor, polyvinyl acetate (PVAc) for the shell, and a solution of PVAc and CaCO_3_ for the core [267]. The hollow and porous nanofibers were obtained after treatment with diluted HCl of the calcined fibers. CaCO_3_ was employed as a pore-forming agent and also for its nucleating effect. In fact, the CaCl_2_ that was formed after the acid treatment was an excellent nucleating agent for CPCs deposition during biomimetic mineralization. 

The pursuit of novel materials to enhance bone regeneration has led to the development of HAp composites with different inorganic materials that can improve the mechanical strength and stiffness, induce the fibroblasts and osteoblast adhesion and proliferation, as well as improve the stimuli-response of the nanocarrier and the electrical conductivity [268,269,270]. Among the new inorganic materials being studied, MXenes, a type of 2D material based on carbides/nitrides of transition metals, has attracted attention due to the presence of multiple functional groups, the large specific surface area, good NIR absorption, and localized surface plasmon resonance that is associated with high photothermal conversion under laser irradiation [268,269,270]. Respecting the last property, the elaboration of MXenes-HAp composites provides an opportunity for preformed thermal ablation of tumor cells while promoting the bone repair of the affected area [269,271]. Based on the good mechanical and biological properties, further investigation should be carried out to determine the potential of electrospun scaffolds that incorporate MXenes-HAp composites for regenerative medicine applications. 

HAp NPs can be encapsulated with high efficiency into electrospun P3HB nanofibers. These showed high mechanical strength, metabolic activity, and mineralization and, consequently, were promising materials for bone tissue regeneration [272]. 

**Figure 17:**
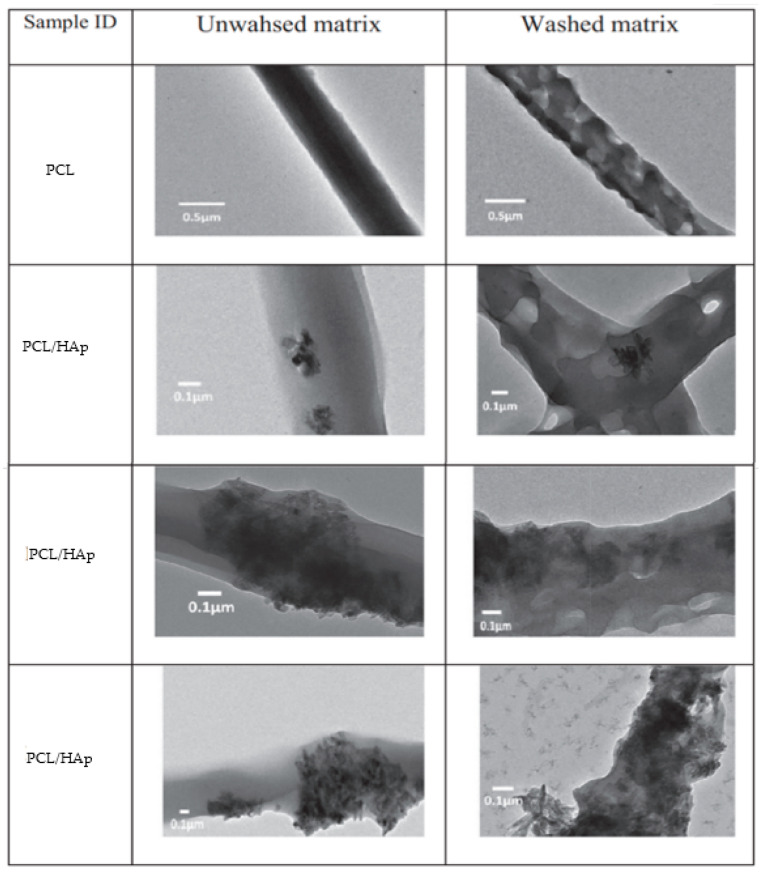
TEM images of unwashed and washed matrices of PCL and PCL/HAp (10%, 30%, and 40% of HAp with respect to PCL) obtained by emulsion electrospinning. Reproduced with permission from [274].

The use of PCL as a matrix polymer for tissue regeneration is increasing, despite the problems derived from the use of organic solvents in conventional electrospinning (i.e., a risk to decrease the capacity of cells to form new tissues in vivo due to the toxicity of not completely removed traces of the solvent), and there is a necessity to reduce the PCL intrinsic hydrophobicity by blending with hydrophilic polymers. The emulsion electrospinning of a PCL/HAp oil-in-water emulsion has been proposed as a viable alternative [273]. Thus, a PCL toluene solution incorporating different percentages of HAp was dispersed in an aqueous phase containing PVA, which was used as a template polymer and removed after washing the electrospun scaffold [274]. TEM micrographs (Figure 17) revealed the confinement of HAp in the PCL matrix. Nevertheless, its presence on the fiber surface was evidenced after water washing. Agglomerates were significant in the preparations with the highest HAp content. Scaffolds having 30% HAp showed a clearly enhanced osteoblast proliferation.

Fiber formation has been studied for the PCL/HAp system, and it was found that the viscoelastic interaction between the dispersed and continuous phases was fundamental [275]. Uniform and coalescence of confined droplets were attained by the optimization of PVA content in the continuous phase. PCL concentration was also relevant to stretch and effectively orient the caged droplets. 

Emulsion electrospinning appears as an ideal process to encapsulate hydrophilic drugs and minimize the burst effect and obtain a long-term sustained release. Clear examples of drug retention of samples prepared by emulsion electrospinning correspond to the encapsulation of lactase in poly(D,L-lactide) (PDLLA) [276], the nerve growth factor (NGF) in copolymers of lactide and ε-caprolactone (PLCL) [277], or bovine serum albumin BSA in PDLLA [278]. 

Laminins are high molecular-weight proteins that form part of the ECM and have an influence on cell differentiation, migration, and adhesion [279]. HAp and laminin have been encapsulated together for the first time by emulsion electrospinning and employing PLCL as a polymer matrix. Fibers showed an appropriate structure and the scaffold revealed good mechanical properties (e.g., Young’s modulus of 37.0 MPa) and enhanced proliferation of fibroblasts [280]. 

Emulsion electrospinning has also been applied to obtain scaffolds based on a PCL-PEO mixture that incorporates the platelet-derived growth factor-BB (PDGF-BB) and different percentages of a HAp/tricalcium phosphate mixture [281]. The release of lysozyme was studied as a model protein. Its interactions with the ceramic component caused a delayed release, a feature that could be avoided with the addition of the cetyltrimethylammonium bromide surfactant. Human MSCs expressed higher levels of osteogenic markers with respect to scaffolds without the growth factor. The technique seems suitable to incorporate growth factors to promote osteoinduction and facilitate bone tissue regeneration. 

The use of emulsifiers may be problematic due to their detrimental effects on the scaffold’s mechanical properties and also because of their leaching at the implantation site. The use of solid particles (Pickering stabilizers) to stabilize the oil–water interface has been proposed as an interesting alternative to conventional emulsifiers. Furthermore, particles can provide new functionalities. The positive effect of HAp NPs to stabilize oil-in-water emulsions has also been reported [282]. Specifically, HAp was used as a stabilizer for the preparation of PCL/HAp scaffolds by emulsion electrospinning [283]. Materials displayed a high osteoblast cell proliferation efficacy and bio-mineralization capacity. 

Scaffolds of PCL-grafted acrylic acid (PCL-*g*-AA) and HAp have been obtained by emulsion electrospinning in absence of an emulsifier [284]. In this case, PVA was added in the water phase as a template polymer. Interestingly, it was found that the carboxylic groups of AA were able to establish interactions with PVA and HAp in such a way that the oil-in-water emulsions were stabilized.

MEW allows the production of scaffolds with an appropriate pore size (ca. 100 μm) to favour cell ingrowth and tissue vascularization and avoid the use of toxic organic solvents, which are two of the main challenges of conventional electrospinning. Works concerning the preparation of melt electrospun written scaffolds constituted by a single thermoplastic polymer are abundant, but these scaffolds can be considered bio-inert and, consequently, without a specific interest in tissue regeneration. Nevertheless, it has recently been described the preparation of 3D PCL/HAp structures by direct writing technology [285]. PCL appears as an ideal polymer due to its flexibility and low melting point. The experimental procedure consisted of the preparation of homogeneous PCL/HAp films by the solvent casting of the corresponding dispersion. The cast samples were then chopped and placed in the syringe barrel of the MEW device. Confocal microscopy images demonstrated continuous cell growth with the highest spreading and bridging for scaffolds incorporating 7% of HAp.

Polymer degradation is a serious restriction of MES that limits the use of samples with a high melting point. Laser melt electrospinning has recently been proposed as a method to minimize degradation due to the use of rapid and uniform CO_2_ laser heating. In this way, PLA/HAp scaffolds have successfully been prepared by this laser MES method, considering that the dispersion and hydrophilicity of fiber mats are carefully investigated [286]. 

**Figure 18:**
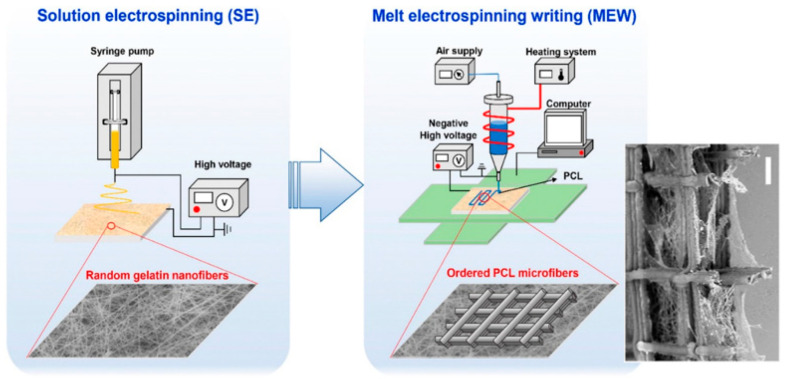
Scheme showing the fabrication of hierarchical structures by combining MEW and conventional electrospinning techniques. The inset shows an SEM image of the multi-layered scaffold (scale bar corresponds to 40 µm). Reproduced with permission from [287].

Hybrid processes combining MEW and solution electrospinning have also been considered since MEW cannot mimic well the structure of the nanoscale ECM. A micro/nano hierarchical structure has, for example, been produced by stacking PCL layers prepared by MEW and electrospun layers of crosslinked Gel nanofibers (Figure 18) [287]. Results pointed out that both cell adhesion and proliferation (Saos-2 cells) and osteoconductivity were improved using the hybrid scaffold. Furthermore, cell penetration was not inhibited by the solution electrospun layers. Mineralized calcium nodules were randomly distributed in all the areas of scaffolds as revealed by alizarin red S staining.

## 6. Perspective and Conclusions

The advances in the development of scaffolds for hard tissue regeneration have been impressive during the last decades. A great understanding of requirements and possibilities has been acquired but, in general, we are still far away from an ideal solution. Nowadays, it appears fundamental the implication and effective collaboration of experts in different scientific areas (e.g., biology, material science, computer science, and electronics). Clinical experience, biological basic principles, and commercial practicality should be combined to obtain optimized designs that involve cells, scaffolds, and active stimuli.

It seems primordial to improve the characteristics of material surfaces in order to develop smart materials with sensory capacities and switchable characteristics according to the changes occurred in the cellular environment. Progress is still necessary to effectively integrate the new constructs into the immune system of the host. Prevention of bacterial infections and biofouling is a vital problem that is not completely solved and that is clearly needed due to the high costs associated with defective implants. The interest in the production of multifunctional and hierarchical scaffolds is evident, as well as the increased applications that are derived. It appears fundamental that there are multiple options that can be given by the combination of different processing techniques, as well as the loading and delivery of bioactive molecules (e.g., inorganic Hap, growth factors, antibacterial, and anti-inflammatory agents, etc.) The selection of appropriate materials, blends, and composites is still challenging, as well as the selection of the appropriate matrix formulation able to mimic specific biological tasks.

Electrospinning offers great variability for processing different materials with highly variable architectures, as well as a high capacity to load a great diversity of active compounds. This high versatility combined with reduced cost and scalability makes electrospinning one technique necessary to build complex architectures and offer systems with the capacity to mimic the ECM environment in living tissues and to design new cancer therapy strategies. In addition, electrospun mats provide shorter hemostasis time and faster recovery than offered by traditional devices, such as sutures. Different designs based on electrospinning are ideal approaches to obtain a local and sustained delivery of bioactive agents with highly different characteristics and properties. Coaxial and emulsion electrospinning of core–shell-structured nanofibers are powerful techniques to obtain control over the release kinetic of entrapped molecules. Multifunctional and stimuli-responsive electrospun patches appear as highly promising systems for local and triggered drug delivery and, consequently, for improved therapeutic efficacy. Great efforts are currently focused to improve both the yield and efficiency of electrospinning (e.g., needle-free, multi-needle, and pulse gas-assisted electrospinning), but probably more improvements are necessary to achieve industrial mass production. It is also critical to produce electrospun scaffolds with improved mechanical properties and be interested in the addition of ceramic nanocompounds (e.g., HAp), and even the application of thermal treatments able to enhance fiber bonding.

Although emulsion and coaxial electrospinning offer alternatives to improve scaffold design and drug loading, each one of them exhibits disadvantages that must be taken into account when elaborating on the scaffolds. For example, the main disadvantage of coaxial electrospinning is the requirement of a complex apparatus and, as two electrospinning tips and two solutions are prepared in order to create the core–sheath fibers, the establishment of the processing parameters for each layer can represent more time. In the case of emulsion electrospinning, sometimes the use of an emulsifier is required to stabilize the emulsion. On another hand, there is not complete control over the inside or outside placement of the pharmaceutical agent in the structure of the fiber, which also conditions the reproducibility of the fibers. 

In addition to the mentioned limitations of the techniques, it appears that additional research concerning in vivo validation is still necessary before the use of these new systems in clinical trials and commercial medical devices.

## Data Availability

Not applicable.

## References

[1] Langer R., Vacanti J.P. (1993). Tissue engineering. Science.

[2] Callister W.D., Rethwisch D.G. (2009). Materials Science and Engineering: An Introduction.

[3] Baroli B. (2009). From natural bone grafts to tissue engineering therapeutics: Brainstorming on pharmaceutical formulative requirements and challenges. J. Pharm. Sci..

[4] Amini A.R., Laurencin C.T., Nukavarapu S.P. (2012). Bone Tissue Engineering: Recent Advances and Challenges. Crit. Rev. Biomed. Eng..

[5] Okike K., Bhattacharyya T. (2006). Trends in the Management of Open Fractures. J. Bone Jt. Surg..

[6] Seebach E., Kubatzky K.F. (2019). Chronic Implant-Related Bone Infections—Can Immune Modulation be a Therapeutic Strategy?. Front. Immunol..

[7] Kohane D.S., Langer R. (2008). Polymeric Biomaterials in Tissue Engineering. Pediatr. Res..

[8] Fuchs R.K., Thompson W.R., Warden S.J. (2019). Bone Repair Biomaterials.

[9] Wang H., Zeng X., Pang L., Wang H., Lin B., Deng Z., Qi E.L.X., Miao N., Wang D., Huang P. (2020). Integrative treatment of anti-tumor/bone repair by combination of MoS2 nanosheets with 3D printed bioactive borosilicate glass scaffolds. Chem. Eng. J..

[10] Chen M., Li Y.-F., Besenbacher F. (2014). Electrospun Nanofibers-Mediated On-Demand Drug Release. Adv. Health Mater..

[11] Bao Z., Gu Z., Xu J., Zhao M., Liu G., Wu J. (2020). Acid-responsive composite hydrogel platform with space-controllable stiffness and calcium supply for enhanced bone regeneration. Chem. Eng. J..

[12] Huang K., Liu G., Gu Z., Wu J. (2020). Tofu as excellent scaffolds for potential bone regeneration. Chin. Chem. Lett..

[13] Stevens M.M. (2008). Biomaterials for bone tissue engineering. Mater. Today.

[14] Bharadwaz A., Jayasuriya A.C. (2020). Recent trends in the application of widely used natural and synthetic polymer nanocomposites in bone tissue regeneration. Mater. Sci. Eng. C.

[15] Roveri N., Iafisco M. (2010). Evolving application of biomimetic nanostructured hydroxyapatite. Nanotechnol. Sci. Appl..

[16] Pang Y., Bao X. (2003). Influence of temperature, ripening time and calcination on the morphology and crystallinity of hydroxyapatite nanoparticles. J. Eur. Ceram. Soc..

[17] Ye F., Guo H., Zhang H., He X. (2010). Polymeric micelle-templated synthesis of hydroxyapatite hollow nanoparticles for a drug delivery system. Acta Biomater..

[18] Xia Z., Liao L., Zhao S. (2009). Synthesis of mesoporous hydroxyapatite using a modified hard-templating route. Mater. Res. Bull..

[19] Zhang C., Yang J., Quan Z., Yang P., Li C., Hou Z., Lin J. (2009). Hydroxyapatite Nano- and Microcrystals with Multiform Morphologies: Controllable Synthesis and Luminescence Properties. Cryst. Growth Des..

[20] Ren F., Leng Y., Ding Y., Wang K. (2013). Hydrothermal growth of biomimetic carbonated apatite nanoparticles with tunable size, morphology and ultrastructure. CrystEngComm.

[21] Sun R., Chen K., Lu Y. (2009). Fabrication and dissolution behavior of hollow hydroxyapatite microspheres intended for controlled drug release. Mater. Res. Bull..

[22] Shum H.C., Bandyopadhyay A., Bose S., Weitz D.A. (2009). Double Emulsion Droplets as Microreactors for Synthesis of Mesoporous Hydroxyapatite. Chem. Mater..

[23] Dou Y., Cai S., Ye X., Xu G., Hu H., Ye X. (2011). Preparation of mesoporous hydroxyapatite films used as biomaterials via sol–gel technology. J. Sol-Gel Sci. Technol..

[24] Lee W.-H., Loo C.-Y., Rohanizadeh R. (2014). A review of chemical surface modification of bioceramics: Effects on protein adsorption and cellular response. Colloids Surfaces B Biointerfaces.

[25] Rechendorff K., Hovgaard M.B., Foss M., Zhdanov V.P., Besenbacher F. (2006). Enhancement of Protein Adsorption Induced by Surface Roughness. Langmuir.

[26] Lee W.-H., Loo C.-Y., Zavgorodniy A.V., Ghadiri M., Rohanizadeh R. (2013). A novel approach to enhance protein adsorption and cell proliferation on hydroxyapatite: Citric acid treatment. RSC Adv..

[27] Holt K.B., Bard A.J. (2005). Interaction of Silver(I) Ions with the Respiratory Chain of *Escherichia coli*: An Electrochemical and Scanning Electrochemical Microscopy Study of the Antimicrobial Mechanism of Micromolar Ag^+^. Biochemistry.

[28] Thangavelu M., Narasimha R.R., Adithan A.A.C., Jong-Hoon K., Parvathaleswara S.T. (2016). Reengineered graft copolymers as a potential alternative for the bone tissue engineering application by inducing osteogenic markers expression and biocompatibility. Colloids Surfaces B Biointerfaces.

[29] Wei J., Liu A., Chen L., Zhang P., Chen X., Jing X. (2009). The Surface Modification of Hydroxyapatite Nanoparticles by the Ring Opening Polymerization of *γ* -Benzyl-L-glutamate *N* -carboxyanhydride. Macromol. Biosci..

[30] Tang Y.-F., Liu J.-G., Wang Z.-L., Wang Y., Cui L.-G., Zhang P.-B., Chen X.-S. (2014). In vivo degradation behavior of porous composite scaffolds of poly(lactide-co-glycolide) and nano-hydroxyapatite surface grafted with poly(L-lactide). Chin. J. Polym. Sci..

[31] Liu Y., Cui H., Zhuang X., Zhang P., Cui Y., Wang X., Wei Y., Chen X. (2013). Nano-hydroxyapatite Surfaces Grafted with Electroactive Aniline Tetramers for Bone-Tissue Engineering. Macromol. Biosci..

[32] Liu Y., Lu Y., Tian X., Cui G., Zhao Y., Yang Q., Yu S., Xing G., Zhang B. (2009). Segmental bone regeneration using an rhBMP-2-loaded gelatin/nanohydroxyapatite/fibrin scaffold in a rabbit model. Biomaterials.

[33] Sotome S., Uemura T., Kikuchi M., Chen J., Itoh S., Tanaka J., Tateishi T., Shinomiya K. (2004). Synthesis and in vivo evaluation of a novel hydroxyapatite/collagen–alginate as a bone filler and a drug delivery carrier of bone morphogenetic protein. Mater. Sci. Eng. C.

[34] Maehara H., Sotome S., Yoshii T., Torigoe I., Kawasaki Y., Sugata Y., Yuasa M., Hirano M., Mochizuki N., Kikuchi M. (2010). Repair of large osteochondral defects in rabbits using porous hydroxyapatite/collagen (HAp/Col) and fibroblast growth factor-2 (FGF-2). J. Orthop. Res..

[35] Phipps M.C., Xu Y., Bellis S.L. (2012). Delivery of Platelet-Derived Growth Factor as a Chemotactic Factor for Mesenchymal Stem Cells by Bone-Mimetic Electrospun Scaffolds. PLoS ONE.

[36] Hwang S.-J., Lee J.-S., Ryu T.-K., Kang R.-H., Jeong K.-Y., Jun D.-R., Koh J.-M., Kim S.-E., Choi S.-W. (2016). Alendronate-modified hydroxyapatite nanoparticles for bone-specific dual delivery of drug and bone mineral. Macromol. Res..

[37] Dou X.-C., Zhu X.-P., Zhou J., Cai H.-Q., Tang J., Li Q.-L. (2011). Minocycline-released hydroxyapatite–gelatin nanocomposite and its cytocompatibility. Vitro Biomed. Mater..

[38] Song W., Yu X., Markel D.C., Shi T., Ren W. (2013). Coaxial PCL/PVA electrospun nanofibers: Osseointegration enhancer and controlled drug release device. Biofabrication.

[39] McNally M.A., Ferguson J.Y., Lau A.C.K., Diefenbeck M., Scarborough M., Ramsden A.J., Atkins B.L. (2016). Single-stage treatment of chronic osteomyelitis with a new absorbable, gentamicin-loaded, calcium sulphate/hydroxyapatite biocomposite. Bone Jt. J..

[40] Ferraz M., Mateus A., Sousa J., Monteiro F. (2007). Nanohydroxyapatite microspheres as delivery system for antibiotics: Release kinetics, antimicrobial activity, and interaction with osteoblasts. J. Biomed. Mater. Res. Part A.

[41] Liu T.-Y., Chen S.-Y., Li J.-H., Liu D.-M. (2006). Study on drug release behaviour of CDHA/chitosan nanocomposites—Effect of CDHA nanoparticles. J. Control. Release.

[42] Rivas M., del Valle L.J., Rodríguez-Rivero A.M., Turon P., Puiggalí J., Alemán C. (2018). Loading of Antibiotic into Biocoated Hydroxyapatite Nanoparticles: Smart Antitumor Platforms with Regulated Release. ACS Biomater. Sci. Eng..

[43] Rivas M., Pelechà M., Franco L., Turon P., Alemán C., del Valle L.J., Puiggalí J. (2019). Incorporation of Chloramphenicol Loaded Hydroxyapatite Nanoparticles into Polylactide. Int. J. Mol. Sci..

[44] Kadkhodaie-Elyaderani A., de Lama-Odría M.D.C., Rivas M., Martínez-Rovira I., Yousef I., Puiggalí J., del Valle L.J. (2022). Medicated Scaffolds Prepared with Hydroxyapatite/Streptomycin Nanoparticles Encapsulated into Polylactide Microfibers. Int. J. Mol. Sci..

[45] Sokolova V.V., Radtke I., Heumann R., Epple M. (2006). Effective transfection of cells with multi-shell calcium phosphate-DNA nanoparticles. Biomaterials.

[46] Welzel T., Radtke I., Meyer-Zaika W., Heumann R., Epple M. (2004). Transfection of cells with custom-made calcium phosphate nanoparticles coated with DNA. J. Mater. Chem..

[47] Zuo G., Wan Y., Meng X., Zhao Q., Ren K., Jia S., Wang J. (2011). Synthesis and characterization of a lamellar hydroxyapatite/DNA nanohybrid. Mater. Chem. Phys..

[48] Revilla-López G., Casanovas J., Bertran O., Turon P., Puiggalí J., Alemán C. (2013). Modeling biominerals formed by apatites and DNA. Biointerphases.

[49] Bertran O., del Valle L.J., Revilla-López G., Chaves G., Cardús L., Casas M.T., Casanovas J., Turon P., Puiggalí J., Alemán C. (2014). Mineralization of DNA into nanoparticles of hydroxyapatite. Dalton Trans..

[50] Litowczenko J., Woźniak-Budych M.J., Staszak K., Wieszczycka K., Jurga S., Tylkowski B. (2021). Milestones and current achievements in development of multifunctional bioscaffolds for medical application. Bioact. Mater..

[51] Vallet-Regí M., Lozano D., González B., Izquierdo-Barba I. (2020). Biomaterials against Bone Infection. Adv. Healthc. Mater..

[52] Bigham A., Hassanzadeh-Tabrizi S., Rafienia M., Salehi H. (2016). Ordered mesoporous magnesium silicate with uniform nanochannels as a drug delivery system: The effect of calcination temperature on drug delivery rate. Ceram. Int..

[53] Ficai A., Marques C., Ferreira J.M., Andronescu E., Ficai D., Sonmez M. (2014). Multifunctional materials for bone cancer treatment. Int. J. Nanomed..

[54] Bigham A., Foroughi F., Rezvani Ghomi E., Rafienia M., Neisiany R.E., Ramakrishna S. (2020). The journey of multifunctional bone scaffolds fabricated from traditional toward modern techniques. Bio-Des. Manuf..

[55] Eliaz N., Metoki N. (2017). Calcium Phosphate Bioceramics: A Review of Their History, Structure, Properties, Coating Technologies and Biomedical Applications. Materials.

[56] Koester K.J., Ager J.W., Ritchie R.O. (2008). The true toughness of human cortical bone measured with realistically short cracks. Nat. Mater..

[57] Chen F.-M., Zhang M., Wu Z.-F. (2010). Toward delivery of multiple growth factors in tissue engineering. Biomaterials.

[58] Tai Y., Banerjee A., Goodrich R., Jin L., Nam J. (2021). Development and Utilization of Multifunctional Polymeric Scaffolds for the Regulation of Physical Cellular Microenvironments. Polymers.

[59] Kolb H.C., Finn M.G., Sharpless K.B. (2001). Click Chemistry: Diverse Chemical Function from a Few Good Reactions. Angew. Chem. Int. Ed..

[60] Zou Y., Zhang L., Yang L., Zhu F., Ding M., Lin F., Wang Z., Li Y. (2018). “Click” chemistry in polymeric scaffolds: Bioactive materials for tissue engineering. J. Control. Release.

[61] Hassan M., Dave K., Chandrawati R., Dehghani F., Gomes V.G. (2019). 3D printing of biopolymer nanocomposites for tissue engineering: Nanomaterials, processing and structure-function relation. Eur. Polym. J..

[62] Viguet-Carrin S., Garnero P., Delmas P.D. (2005). The role of collagen in bone strength. Osteoporos. Int..

[63] Kołodziejska B., Kaflak A., Kolmas J. (2020). Biologically Inspired Collagen/Apatite Composite Biomaterials for Potential Use in Bone Tissue Regeneration—A Review. Materials.

[64] Cui F.-Z., Li Y., Ge J. (2007). Self-assembly of mineralized collagen composites. Mater. Sci. Eng. R Rep..

[65] Cunniffe G.M., Dickson G.R., Partap S., Stanton K.T., O’Brien F.J. (2010). Development and characterisation of a collagen nano-hydroxyapatite composite scaffold for bone tissue engineering. J. Mater. Sci. Mater. Med..

[66] Fukui N., Sato T., Kuboki Y., Aoki H. (2008). Bone tissue reaction of nano-hydroxyapatite/collagen composite at the early stage of implantation. Bio-Medical. Mater. Eng..

[67] Nishikawa T., Masuno K., Tominaga K., Koyama Y., Yamada T., Takakuda K., Kikuchi M., Tanaka J., Tanaka A. (2005). Bone Repair Analysis in a Novel Biodegradable Hydroxyapatite/Collagen Composite Implanted in Bone. Implant. Dent..

[68] Kikuchi M. (2013). Hydroxyapatite/Collagen Bone-Like Nanocomposite. Biol. Pharm. Bull..

[69] Díaz A., Puiggalí J. (2017). Hydrogels for Biomedical Applications: Cellulose, Chitosan, and Protein/Peptide Derivatives. Gels.

[70] Collins M.N., Birkinshaw C. (2013). Hyaluronic acid based scaffolds for tissue engineering—A review. Carbohydr. Polym..

[71] Kawano M., Ariyoshi W., Iwanaga K., Okinaga T., Habu M., Yoshioka I., Tominaga K., Nishihara T. (2011). Mechanism involved in enhancement of osteoblast differentiation by hyaluronic acid. Biochem. Biophys. Res. Commun..

[72] Sall I., Férard G. (2007). Comparison of the sensitivity of 11 crosslinked hyaluronic acid gels to bovine testis hyaluronidase. Polym. Degrad. Stab..

[73] Chang Y.-L., Hsieh C.-Y., Yeh C.-Y., Lin F.-H. (2019). The Development of Gelatin/Hyaluronate Copolymer Mixed with Calcium Sulfate, Hydroxyapatite, and Stromal-Cell-Derived Factor-1 for Bone Regeneration Enhancement. Polymers.

[74] Choi S., Lee J.S., Shin J., Lee M.S., Kang D., Hwang N.S., Lee H., Yang H.S., Cho S.-W. (2020). Osteoconductive hybrid hyaluronic acid hydrogel patch for effective bone formation. J. Control. Release.

[75] Wenz A., Borchers K., Tovar G.E.M., Kluger P.J. (2017). Bone matrix production in hydroxyapatite-modified hydrogels suitable for bone bioprinting. Biofabrication.

[76] Khor E. (2014). Chitin: Fulfilling a Biomaterials Promise.

[77] Yamaguchi I., Tokuchi K., Fukuzaki H., Koyama Y., Takakuda K., Monma H., Tanaka J. (2001). Preparation and microstructure analysis of chitosan/hydroxyapatite nanocomposites. J. Biomed. Mater. Res..

[78] Ito M. (1991). In vitro properties of a chitosan-bonded hydroxyapatite bone-filling paste. Biomaterials.

[79] Ramesh N., Moratti S.C., Dias G.J. (2018). Hydroxyapatite-polymer biocomposites for bone regeneration: A review of current trends. J. Biomed. Mater. Res. Part B Appl. Biomater..

[80] Dhivya S., Saravanan S., Sastry T.P., Selvamurugan N. (2015). Nanohydroxyapatite-reinforced chitosan composite hydrogel for bone tissue repair in vitro and in vivo. J. Nanobiotechnol..

[81] Biazar E., Heidari Keshel S., Tavirani M.R., Jahandideh R. (2015). Bone reconstruction in rat calvarial defects by chitosan/hydroxyapatite nanoparticles scaffold loaded with unrestricted somatic stem cells. Artif. Cells Nanomed. Biotechnol..

[82] Ma X.-Y., Feng Y.-F., Ma Z.-S., Li X., Wang J., Wang L., Lei W. (2014). The promotion of osteointegration under diabetic conditions using chitosan/hydroxyapatite composite coating on porous titanium surfaces. Biomaterials.

[83] Stanford E.C.C. (1881). Improvements in the Manufacture of Useful Products from Seaweeds. British Patent.

[84] Tampieri A., Sandri M., Landi E., Celotti G., Roveri N., Mattioli-Belmonte M., Virgili L., Gabbanelli F., Biagini G. (2005). HA/alginate hybrid composites prepared through bio-inspired nucleation. Acta Biomater..

[85] Rajkumar M., Meenakshisundaram N., Rajendran V. (2011). Development of nanocomposites based on hydroxyapatite/sodium alginate: Synthesis and characterisation. Mater. Charact..

[86] Jorfi M., Foster E.J. (2014). Recent advances in nanocellulose for biomedical applications. J. Appl. Polym. Sci..

[87] Fu L., Zhang J., Yang G. (2013). Present status and applications of bacterial cellulose-based materials for skin tissue repair. Carbohydr. Polym..

[88] Petersen N., Gatenholm P. (2011). Bacterial cellulose-based materials and medical devices: Current state and perspectives. Appl. Microbiol. Biotechnol..

[89] Aravamudhan A., Ramos D.M., Nip J., Harmon M.D., James R., Deng M., Laurencin C.T., Yu X., Kumbar S.G. (2013). Cellulose and Collagen Derived Micro-Nano Structured Scaffolds for Bone Tissue Engineering. J. Biomed. Nanotechnol..

[90] Zimmermann K.A., LeBlanc J.M., Sheets K.T., Fox R.W., Gatenholm P. (2011). Biomimetic design of a bacterial cellulose/hydroxyapatite nanocomposite for bone healing applications. Mater. Sci. Eng. C.

[91] Balla E., Daniilidis V., Karlioti G., Kalamas T., Stefanidou M., Bikiaris N.D., Vlachopoulos A., Koumentakou I., Bikiaris D.N. (2021). Poly(lactic Acid): A Versatile Biobased Polymer for the Future with Multifunctional Properties—From Monomer Synthesis, Polymerization Techniques and Molecular Weight Increase to PLA Applications. Polymers.

[92] Elmowafy E.M., Tiboni M., Soliman M.E. (2019). Biocompatibility, biodegradation and biomedical applications of poly(lactic acid)/poly(lactic-co-glycolic acid) micro and nanoparticles. J. Pharm. Investig..

[93] Wiegand T., Karr J., Steinkruger J.D., Hiebner K., Simetich B., Beatty M., Redepenning J. (2008). Reconstruction of Anorganic Mammalian Bone by Surface-Initiated Polymerization of L-Lactide. Chem. Mater..

[94] Wiegand T., Hiebner K., Gauza L., Schwartz C., Song Z., Miller S., Zacharias N., Wooley P.H., Redepenning J. (2014). Biomimetic composites by surface-initiated polymerization of cyclic lactones at anorganic bone: Preparation and in vitro evaluation of osteoblast and osteoclast competence. J. Biomed. Mater. Res. Part A.

[95] Hong Z., Zhang P., He C., Qiu X., Liu A., Chen L., Zhu X., Jing X. (2005). Nano-composite of poly(L-lactide) and surface grafted hydroxyapatite: Mechanical properties and biocompatibility. Biomaterials.

[96] Qiu X., Hong Z., Hu J., Chen L., Chen X., Jing X. (2005). Hydroxyapatite Surface Modified by L-Lactic Acid and Its Subsequent Grafting Polymerization of L-Lactide. Biomacromolecules.

[97] Zhang P., Hong Z., Yu T., Chen X., Jing X. (2009). In vivo mineralization and osteogenesis of nanocomposite scaffold of poly(lactide-co-glycolide) and hydroxyapatite surface-grafted with poly(l-lactide). Biomaterials.

[98] Wei G., Ma P.X. (2004). Structure and properties of nano-hydroxyapatite/polymer composite scaffolds for bone tissue engineering. Biomaterials.

[99] Wei G., Ma P.X. (2006). Macroporous and nanofibrous polymer scaffolds and polymer/bone-like apatite composite scaffolds generated by sugar spheres. J. Biomed. Mater. Res. Part A.

[100] He C., Jin X., Ma P.X. (2014). Calcium phosphate deposition rate, structure and osteoconductivity on electrospun poly(l-lactic acid) matrix using electrodeposition or simulated body fluid incubation. Acta Biomater..

[101] Huang Y.X., Ren J., Chen C., Ren T.B., Zhou X.Y. (2008). Preparation and Properties of Poly(lactide-co-glycolide) (PLGA)/ Nano-Hydroxyapatite (NHA) Scaffolds by Thermally Induced Phase Separation and Rabbit MSCs Culture on Scaffolds. J. Biomater. Appl..

[102] Johnson R., Ding Y., Nagiah N., Monnet E., Tan W. (2018). Coaxially-structured fibres with tailored material properties for vascular graft implant. Mater. Sci. Eng. C.

[103] Chopra V., Thomas J., Sharma A., Panwar V., Kaushik S., Sharma S., Porwal K., Kulkarni C., Rajput S., Singh H. (2020). Synthesis and Evaluation of a Zinc Eluting rGO/Hydroxyapatite Nanocomposite Optimized for Bone Augmentation. ACS Biomater. Sci. Eng..

[104] Visakh P.M. (2015). Polyhydroxyalkanoate (PHA) Based Blends, Composites and Nanocomposites.

[105] Chen G.-Q., Wu Q. (2005). The application of polyhydroxyalkanoates as tissue engineering materials. Biomaterials.

[106] Sudesh K., Abe H., Doi Y. (2000). Synthesis, structure and properties of polyhydroxyalkanoates: Biological polyesters. Prog. Polym. Sci..

[107] Cheng S., Chen G., Leski M., Zou B., Wang Y., Wu Q. (2006). The effect of D,L-β-hydroxybutyric acid on cell death and proliferation in L929 cells. Biomaterials.

[108] Kushwah B.S., Kushwah A.V.S., Singh V. (2016). RETRACTED ARTICLE: Towards understanding polyhydroxyalkanoates and their use. J. Polym. Res..

[109] Brigham C.J., Sinskey A.J. (2012). Applications of Polyhydroxyalkanoates in the Medical Industry. Int. J. Biotechnol. Wellness Ind..

[110] Hong S.-G., Hsu H.-W., Ye M.-T. (2013). Thermal properties and applications of low molecular weight polyhydroxybutyrate. J. Therm. Anal. Calorim..

[111] Lopes P.P., Garcia M.P., Fernandes M.H., Fernandes M.H.V. (2013). Acrylic formulations containing bioactive and biodegradable fillers to be used as bone cements: Properties and biocompatibility assessment. Mater. Sci. Eng. C.

[112] Sadat-Shojai M., Khorasani M.-T., Jamshidi A., Irani S. (2013). Nano-hydroxyapatite reinforced polyhydroxybutyrate composites: A comprehensive study on the structural and in vitro biological properties. Mater. Sci. Eng. C.

[113] Shishatskaya E.I., Khlusov I.A., Volova T.G. (2006). A hybrid PHB–hydroxyapatite composite for biomedical application: Production, in vitro and in vivo investigation. J. Biomater. Sci. Polym. Ed..

[114] Ramier J., Grande D., Bouderlique T., Stoilova O., Manolova N., Rashkov I., Langlois V., Albanese P., Renard E. (2014). From design of bio-based biocomposite electrospun scaffolds to osteogenic differentiation of human mesenchymal stromal cells. J. Mater. Sci. Mater. Med..

[115] Bernd H.E., Kunze C., Freier T., Sternberg K., Kramer S., Behrend D., Prall F., Donat M., Kramp B. (2009). Poly(3-hydroxybutyrate) (PHB) patches for covering anterior skull base defects—An animal study with minipigs. Acta Oto-Laryngol..

[116] Gredes T., Gedrange T., Hinüber C., Gelinsky M., Kunert-Keil C. (2015). Histological and molecular-biological analyses of poly(3-hydroxybutyrate) (PHB) patches for enhancement of bone regeneration. Ann. Anat.—Anat. Anz..

[117] Alves E.G.L., Rezende C.M.D.F., Serakides R., Pereira M.D.M., Rosado I.R. (2011). Orthopedic implant of a polyhydroxybutyrate (PHB) and hydroxyapatite composite in cats. J. Feline Med. Surg..

[118] Celarek A., Kraus T., Tschegg E.K., Fischerauer S.F., Stanzl-Tschegg S., Uggowitzer P.J., Weinberg A.M. (2012). PHB, crystalline and amorphous magnesium alloys: Promising candidates for bioresorbable osteosynthesis implants?. Mater. Sci. Eng. C.

[119] Doyle C., Tanner E.T., Bonfield W. (1991). In vitro and in vivo evaluation of polyhydroxybutyrate and of polyhydroxybutyrate reinforced with hydroxyapatite. Biomaterials.

[120] Ni J., Wang M. (2002). In vitro evaluation of hydroxyapatite reinforced polyhydroxybutyrate composite. Mater. Sci. Eng. C.

[121] Galego N., Rozsa C., Sánchez R., Fung J., Vázquez A., Santo Tomás J. (2000). Characterization and application of poly(β-hydroxyalkanoates) family as composite biomaterials. Polym. Test..

[122] Guo B., Ma P.X. (2018). Conducting Polymers for Tissue Engineering. Biomacromolecules.

[123] Fujii E., Ohkubo M., Tsuru K., Hayakawa S., Osaka A., Kawabata K., Bonhomme C., Babonneau F. (2006). Selective protein adsorption property and characterization of nano-crystalline zinc-containing hydroxyapatite. Acta Biomater..

[124] Lee H., Dellatore S.M., Miller W.M., Messersmith P.B. (2007). Mussel-Inspired Surface Chemistry for Multifunctional Coatings. Science.

[125] Zhang S., Gangal G., Uludağ H. (2007). ‘Magic bullets’ for bone diseases: Progress in rational design of bone-seeking medicinal agents. Chem. Soc. Rev..

[126] Iafisco M., Palazzo B., Falini G., di Foggia M., Bonora S., Nicolis S., Casella L., Roveri N. (2008). Adsorption and Conformational Change of Myoglobin on Biomimetic Hydroxyapatite Nanocrystals Functionalized with Alendronate. Langmuir.

[127] Schuessele A., Mayr H., Tessmar J., Goepferich A. (2009). Enhanced bone morphogenetic protein-2 performance on hydroxyapatite ceramic surfaces. J. Biomed. Mater. Res. Part A.

[128] Kandori K., Oda S., Tsuyama S. (2008). Effects of Pyrophosphate Ions on Protein Adsorption onto Calcium Hydroxyapatite. J. Phys. Chem. B.

[129] Szurkowska K., Laskus A., Kolmas J. (2018). Hydroxyapatite—Advances in Composite Nanomaterials, Biomedical Applications and Its Technological Facets.

[130] Kolmas J., Krukowski S., Laskus A., Jurkitewicz M. (2016). Synthetic hydroxyapatite in pharmaceutical applications. Ceram. Int..

[131] Turon P., del Valle L.J., Alemán C., Puiggalí J. (2018). Biopolymer Grafting Applications.

[132] Lian X., Mao K., Liu X., Wang X., Cui F. (2015). In Vivo Osteogenesis of Vancomycin Loaded Nanohydroxyapatite/Collagen/Calcium Sulfate Composite for Treating Infectious Bone Defect Induced by Chronic Osteomyelitis. J. Nanomater..

[133] Suvannapruk W., Thammarakcharoen F., Phanpiriya P., Suwanprateeb J. (2013). Development of Antibiotics Impregnated Nanosized Silver Phosphate-Doped Hydroxyapatite Bone Graft. J. Nanomater..

[134] Tesema Y., Raghavan D., Stubbs J. (2004). Bone cell viability on collagen immobilized poly(3-hydroxybutrate-co-3-hydroxyvalerate) membrane: Effect of surface chemistry. J. Appl. Polym. Sci..

[135] Chen X., Zhang X., Zhu Y., Zhang J., Hu P. (2003). Surface Modification of Polyhydroxyalkanoates by Ion Implantation. Characterization and Cytocompatibility Improvement. Polym. J..

[136] Zuo C., Huang Y., Bajis R., Sahih M., Li Y.-P., Dai K., Zhang X. (2012). Osteoblastogenesis regulation signals in bone remodeling. Osteoporos. Int..

[137] Tafazoli Moghadam E., Yazdanian M., Alam M., Tebyanian H., Tafazoli A., Tahmasebi E., Ranjbar R., Yazdanian A., Seifalian A. (2021). Current natural bioactive materials in bone and tooth regeneration in dentistry: A comprehensive overview. J. Mater. Res. Technol..

[138] Kaida K., Honda Y., Hashimoto Y., Tanaka M., Baba S. (2015). Application of Green Tea Catechin for Inducing the Osteogenic Differentiation of Human Dedifferentiated Fat Cells In Vitro. Int. J. Mol. Sci..

[139] Bhattacharya S., Chandra S., Chatterjee P., Dey P. (2012). Evaluation of anti-inflammatory effects of green tea and black tea: A comparative in vitro study. J. Adv. Pharm. Technol. Res..

[140] Hengge R. (2019). Targeting Bacterial Biofilms by the Green Tea Polyphenol EGCG. Molecules.

[141] Boonyagul S., Banlunara W., Sangvanich P., Thunyakitpisal P. (2013). Effect of acemannan, an extracted polysaccharide from Aloe vera, on BMSCs proliferation, differentiation, extracellular matrix synthesis, mineralization, and bone formation in a tooth extraction model. Odontology.

[142] Le Van C., Thu H.P.T., Sangvanich P., Chuenchompoonut V., Thunyakitpisal P. (2020). Acemannan induces rapid early osseous defect healing after apical surgery: A 12-month follow-up of a randomized controlled trial. J. Dent. Sci..

[143] Xie Y., Sun W., Yan F., Liu H., Deng Z., Cai L. (2019). Icariin-loaded porous scaffolds for bone regeneration through the regulation of the coupling process of osteogenesis and osteoclastic activity. Int. J. Nanomed. Ume.

[144] Lai Y., Cao H., Wang X., Chen S., Zhang M., Wang N., Yao Z., Dai Y., Xie X., Zhang P. (2018). Porous composite scaffold incorporating osteogenic phytomolecule icariin for promoting skeletal regeneration in challenging osteonecrotic bone in rabbits. Biomaterials.

[145] Ahangari N., Kargozar S., Ghayour-Mobarhan M., Baino F., Pasdar A., Sahebkar A., Ferns G.A.A., Kim H., Mozafari M. (2019). Curcumin in tissue engineering: A traditional remedy for modern medicine. BioFactors.

[146] Li Y., Zhang Z.-Z. (2018). Sustained curcumin release from PLGA microspheres improves bone formation under diabetic conditions by inhibiting the reactive oxygen species production. Drug Des. Dev. Ther. Ume..

[147] Sarkar N., Bose S. (2019). Liposome-Encapsulated Curcumin-Loaded 3D Printed Scaffold for Bone Tissue Engineering. ACS Appl. Mater. Interfaces.

[148] Zhai J.-L., Weng X.-S., Wu Z.-H., Guo S.-G. (2016). Effect of Resveratrol on Preventing Steroid-induced Osteonecrosis in a Rabbit Model. Chin. Med. J..

[149] Casarin R., Casati M., Pimentel S., Cirano F., Algayer M., Pires P., Ghiraldini B., Duarte P., Ribeiro F. (2014). Resveratrol improves bone repair by modulation of bone morphogenetic proteins and osteopontin gene expression in rats. Int. J. Oral Maxillofac. Surg..

[150] Wang C.-C., Wang C.-H., Chen H.-C., Cherng J.-H., Chang S.-J., Wang Y.-W., Chang A., Yeh J.-Z., Huang Y.-H., Liu C.-C. (2018). Combination of resveratrol-containing collagen with adipose stem cells for craniofacial tissue-engineering applications. Int. Wound J..

[151] Poornima B., Korrapati P.S. (2017). Fabrication of chitosan-polycaprolactone composite nanofibrous scaffold for simultaneous delivery of ferulic acid and resveratrol. Carbohydr. Polym..

[152] Notodihardjo F.Z., Kakudo N., Kushida S., Suzuki K., Kusumoto K. (2012). Bone regeneration with BMP-2 and hydroxyapatite in critical-size calvarial defects in rats. J. Cranio-Maxillofac. Surg..

[153] Feito M.J., Serrano M.C., Oñaderra M., Matesanz M.C., Sánchez-Salcedo S., Arcos D., Vallet-Regí M., Portolés M.T. (2016). Effects of immobilized VEGF on endothelial progenitor cells cultured on silicon substituted and nanocrystalline hydroxyapatites. RSC Adv..

[154] Rozen N., Bick T., Bajayo A., Shamian B., Schrift-Tzadok M., Gabet Y., Yayon A., Bab I., Soudry M., Lewinson D. (2009). Transplanted blood-derived endothelial progenitor cells (EPC) enhance bridging of sheep tibia critical size defects. Bone.

[155] Tsurushima H., Marushima A., Suzuki K., Oyane A., Sogo Y., Nakamura K., Matsumura A., Ito A. (2010). Enhanced bone formation using hydroxyapatite ceramic coated with fibroblast growth factor-2. Acta Biomater..

[156] Sun T., Qu Y., Cui W., Yang L., Ji Y., Yu W., Navinduth R., Shao Z., Yang H., Guo X. (2017). Evaluation of osteogenic inductivity of a novel BMP2-mimicking peptide P28 and P28-containing bone composite. J. Biomed. Mater. Res. Part A.

[157] Huang Y., Ren J., Ren T., Gu S., Tan Q., Zhang L., Lv K., Pan K., Jiang X. (2010). Bone marrow stromal cells cultured on poly (lactide-co-glycolide)/nano-hydroxyapatite composites with chemical immobilization of Arg-Gly-Asp peptide and preliminary bone regeneration of mandibular defect thereof. J. Biomed. Mater. Res. Part A.

[158] Kim H.K., Kim J.H., Park D.S., Park K.S., Kang S.S., Lee J.S., Jeong M.H., Yoon T.R. (2012). Osteogenesis induced by a bone forming peptide from the prodomain region of BMP-7. Biomaterials.

[159] Reneker D.H., Chun I. (1996). Nanometre diameter fibres of polymer, produced by electrospinning. Nanotechnology.

[160] Reneker D.H., Yarin A.L., Fong H., Koombhongse S. (2000). Bending instability of electrically charged liquid jets of polymer solutions in electrospinning. J. Appl. Phys..

[161] Frenot A., Chronakis I.S. (2003). Polymer nanofibers assembled by electrospinning. Curr. Opin. Colloid Interface Sci..

[162] Dzenis Y. (2004). Spinning Continuous Fibers for Nanotechnology. Science.

[163] Li D., Xia Y. (2004). Electrospinning of Nanofibers: Reinventing the Wheel?. Adv. Mater..

[164] Jayaraman K., Kotaki M., Zhang Y., Mo X., Ramakrishna S. (2004). Recent advances in polymer nanofibers. J. Nanosci. Nanotechnol..

[165] Dhakate S.R., Singla B., Uppal M., Mathur R.B. (2010). Effect of processing parameters on morphology and thermal properties of electrospun polycarbonate nanofibers. Adv. Mater. Lett..

[166] Sharma S. (2013). Ferrolectric Nanofibers: Principle, Processing and Applications. Adv. Mater. Lett..

[167] Dersch R., Steinhart M., Boudriot U., Greiner A., Wendorff J.H. (2005). Nanoprocessing of polymers: Applications in medicine, sensors, catalysis, photonics. Polym. Adv. Technol..

[168] Chronakis I.S. (2005). Novel nanocomposites and nanoceramics based on polymer nanofibers using electrospinning process—A review. J. Mater. Process. Technol..

[169] Deitzel J.M., Kleinmeyer J., Harris D., Beck Tan N.C. (2001). The effect of processing variables on the morphology of electrospun nanofibers and textiles. Polymer.

[170] Llorens E., Armelin E., del Mar Pérez-Madrigal M., del Valle L.J., Alemán C., Puiggalí J. (2013). Nanomembranes and Nanofibers from Biodegradable Conducting Polymers. Polymers.

[171] Sankar S., Sharma C.S., Rath S.N., Ramakrishna S. (2017). Electrospun Fibers for Recruitment and Differentiation of Stem Cells in Regenerative Medicine. Biotechnol. J..

[172] Wang K., Liu L., Xie J., Shen L., Tao J., Zhu J. (2018). Facile Strategy to Generate Aligned Polymer Nanofibers: Effects on Cell Adhesion. ACS Appl. Mater. Interfaces.

[173] Denchai A., Tartarini D., Mele E. (2018). Cellular Response to Surface Morphology: Electrospinning and Computational Modeling. Front. Bioeng. Biotechnol..

[174] Badami A.S., Kreke M.R., Thompson M.S., Riffle J.S., Goldstein A.S. (2006). Effect of fiber diameter on spreading, proliferation, and differentiation of osteoblastic cells on electrospun poly(lactic acid) substrates. Biomaterials.

[175] Chen Y., Shafiq M., Liu M., Morsi Y., Mo X. (2020). Advanced fabrication for electrospun three-dimensional nanofiber aerogels and scaffolds. Bioact. Mater..

[176] Blakeney B.A., Tambralli A., Anderson J.M., Andukuri A., Lim D.-J., Dean D.R., Jun H.-W. (2011). Cell infiltration and growth in a low density, uncompressed three-dimensional electrospun nanofibrous scaffold. Biomaterials.

[177] Chainani A., Hippensteel K.J., Kishan A., Garrigues N.W., Ruch D.S., Guilak F., Little D. (2013). Multilayered Electrospun Scaffolds for Tendon Tissue Engineering. Tissue Eng. Part A.

[178] Sun B., Li J., Liu W., Aqeel B.M., El-Hamshary H., Al-Deyab S.S., Mo X. (2015). Fabrication and characterization of mineralized P(LLA-CL)/SF three-dimensional nanoyarn scaffolds. Iran. Polym. J..

[179] Kim T.G., Chung H.J., Park T.G. (2008). Macroporous and nanofibrous hyaluronic acid/collagen hybrid scaffold fabricated by concurrent electrospinning and deposition/leaching of salt particles. Acta Biomater..

[180] Shim I.K., Suh W.H., Lee S.Y., Lee S.H., Heo S.J., Lee M.C., Lee S.J. (2009). Chitosan nano-/microfibrous double-layered membrane with rolled-up three-dimensional structures for chondrocyte cultivation. J. Biomed. Mater. Res. Part A.

[181] Sampath Kumar T.S., Yogeshwar Chakrapani V. (2018). Electrospun 3D Scaffolds for Tissue Regeneration. Cutting-Edge Enabling Technologies for Regenerative Medicine. Advances in Experimental Medicine and Biology.

[182] Chen Y., Yu Z., Meng X., Li H., Sun X., He D., Zhang Y., Zhang Z. (2022). Localized surface plasmon resonance improves transdermal photodynamic therapy of hypertrophic scars. Nano Res..

[183] Vyas C., Ates G., Aslan E., Hart J., Huang B., Bartolo P. (2020). Three-Dimensional Printing and Electrospinning Dual-Scale Polycaprolactone Scaffolds with Low-Density and Oriented Fibers to Promote Cell Alignment. 3D Print. Addit. Manuf..

[184] Guo Y., Huang J., Fang Y., Huang H., Wu J. (2022). 1D, 2D, and 3D scaffolds promoting angiogenesis for enhanced wound healing. Chem. Eng. J..

[185] Shafiq M., Zhang Q., Zhi D., Wang K., Kong D., Kim D.-H., Kim S.H. (2018). In Situ Blood Vessel Regeneration Using SP (Substance P) and SDF (Stromal Cell–Derived Factor)-1α Peptide Eluting Vascular Grafts. Arter. Thromb. Vasc. Biol..

[186] Gao X., Han S., Zhang R., Liu G., Wu J. (2019). Progress in electrospun composite nanofibers: Composition, performance and applications for tissue engineering. J. Mater. Chem. B.

[187] Ghosh S.K., Adhikary P., Jana S., Biswas A., Sencadas V., Gupta S.D., Tudu B., Mandal D. (2017). Electrospun gelatin nanofiber based self-powered bio-e-skin for health care monitoring. Nano Energy.

[188] Zhang J., Zhao Y.-T., Hu P.-Y., Liu J.-J., Liu X.-F., Hu M., Cui Z., Wang N., Niu Z., Xiang H.-F. (2020). Laparoscopic electrospinning for in situ hemostasis in minimally invasive operation. Chem. Eng. J..

[189] Hutmacher D.W., Dalton P.D. (2011). Melt Electrospinning. Chem. Asian J..

[190] Bachs-Herrera A., Yousefzade O., del Valle L., Puiggali J. (2021). Melt Electrospinning of Polymers: Blends, Nanocomposites, Additives and Applications. Appl. Sci..

[191] Brown T.D., Dalton P.D., Hutmacher D.W. (2016). Melt electrospinning today: An opportune time for an emerging polymer process. Prog. Polym. Sci..

[192] Zhang L.-H., Duan X.-P., Yan X., Yu M., Ning X., Zhao Y., Long Y.-Z. (2016). Recent advances in melt electrospinning. RSC Adv..

[193] Góra A., Sahay R., Thavasi V., Ramakrishna S. (2011). Melt-Electrospun Fibers for Advances in Biomedical Engineering, Clean Energy, Filtration, and Separation. Polym. Rev..

[194] Dalton P.D. (2017). Melt electrowriting with additive manufacturing principles. Curr. Opin. Biomed. Eng..

[195] Castilho M., Feyen D., Flandes-Iparraguirre M., Hochleitner G., Groll J., Doevendans P.A.F., Vermonden T., Ito K., Sluijter J.P.G., Malda J. (2017). Melt Electrospinning Writing of Poly-Hydroxymethylglycolide-*co*-ε-Caprolactone-Based Scaffolds for Cardiac Tissue Engineering. Adv. Healthc. Mater..

[196] Sun Z., Zussman E., Yarin A.L., Wendorff J.H., Greiner A. (2003). Compound Core–Shell Polymer Nanofibers by Co-Electrospinning. Adv. Mater..

[197] Huang J., Cao Y., Huang Z., Imbraguglio S.A., Wang Z., Peng X., Guo Z. (2016). Comparatively Thermal and Crystalline Study of Poly(methyl-methacrylate)/Polyacrylonitrile Hybrids: Core-Shell Hollow Fibers, Porous Fibers, and Thin Films. Macromol. Mater. Eng..

[198] Elahi M.F., Lu W., Guoping G., Khan F. (2013). Core-shell fibers for biomedical applications—A review. J. Bioeng. Biomed. Sci..

[199] Han D., Steckl A.J. (2013). Triaxial Electrospun Nanofiber Membranes for Controlled Dual Release of Functional Molecules. ACS Appl. Mater. Interfaces.

[200] Yang H.-S., Lee B.-S., You B.-C., Sohn H.-J., Yu W.-R. (2014). Fabrication of carbon nanofibers with Si nanoparticle-stuffed cylindrical multi-channels via coaxial electrospinning and their anodic performance. RSC Adv..

[201] Rahimi M., Mokhtari J. (2018). Fabrication of thermo-regulating hexadecane-polyurethane core-shell composite nanofibrous mat as advanced technical layer: Effect of coaxial nozzle geometry. J. Ind. Text..

[202] Yu D.-G., Branford-White C., Bligh S.W.A., White K., Chatterton N.P., Zhu L.-M. (2011). Improving Polymer Nanofiber Quality Using a Modified Co-axial Electrospinning Process. Macromol. Rapid Commun..

[203] Xu Y., Li J.-J., Yu D.-G., Williams G.R., Yang J.-H., Wang X. (2017). Influence of the drug distribution in electrospun gliadin fibers on drug-release behavior. Eur. J. Pharm. Sci..

[204] Wang Q., Yu D.-G., Zhang L.-L., Liu X.-K., Deng Y.-C., Zhao M. (2017). Electrospun hypromellose-based hydrophilic composites for rapid dissolution of poorly water-soluble drug. Carbohydr. Polym..

[205] Nguyen T.T.T., Chung O.H., Park J.S. (2011). Coaxial electrospun poly(lactic acid)/chitosan (core/shell) composite nanofibers and their antibacterial activity. Carbohydr. Polym..

[206] Yi F., LaVan D.A. (2008). Poly(glycerol sebacate) Nanofiber Scaffolds by Core/Shell Electrospinning. Macromol. Biosci..

[207] Huang Z.-M., Zhang Y., Ramakrishna S. (2005). Double-layered composite nanofibers and their mechanical performance. J. Polym. Sci. Part B Polym. Phys..

[208] He C.-L., Huang Z.-M., Han X.-J. (2009). Fabrication of drug-loaded electrospun aligned fibrous threads for suture applications. J. Biomed. Mater. Res. Part A.

[209] Sill T.J., von Recum H.A. (2008). Electrospinning: Applications in drug delivery and tissue engineering. Biomaterials.

[210] Xiaoqiang L., Yan S., Rui C., Chuanglong H., Hongsheng W., Xiumei M. (2008). Fabrication and properties of core-shell structure P(LLA-CL) nanofibers by coaxial electrospinning. J. Appl. Polym. Sci..

[211] Llorens E., Ibañez H., del Valle L.J., Puiggalí J. (2015). Biocompatibility and drug release behavior of scaffolds prepared by coaxial electrospinning of poly(butylene succinate) and polyethylene glycol. Mater. Sci. Eng. C.

[212] Khalf A., Singarapu K., Madihally S.V. (2015). Cellulose acetate core–shell structured electrospun fiber: Fabrication and characterization. Cellulose.

[213] Lallave M., Bedia J., Ruiz-Rosas R., Rodríguez-Mirasol J., Cordero T., Otero J.C., Marquez M., Barrero A., Loscertales I.G. (2007). Filled and Hollow Carbon Nanofibers by Coaxial Electrospinning of Alcell Lignin without Binder Polymers. Adv. Mater..

[214] Ou K.-L., Chen C.-S., Lin L.-H., Lu J.-C., Shu Y.-C., Tseng W.-C., Yang J.-C., Lee S.-Y., Chen C.-C. (2011). Membranes of epitaxial-like packed, super aligned electrospun micron hollow poly(l-lactic acid) (PLLA) fibers. Eur. Polym. J..

[215] Pakravan M., Heuzey M.-C., Ajji A. (2012). Core–Shell Structured PEO-Chitosan Nanofibers by Coaxial Electrospinning. Biomacromolecules.

[216] Zhou F.-L., Hubbard P.L., Eichhorn S.J., Parker G.J.M. (2012). Coaxially Electrospun Axon-Mimicking Fibers for Diffusion Magnetic Resonance Imaging. ACS Appl. Mater. Interfaces.

[217] Ji X., Su Z., Wang P., Ma G., Zhang S. (2014). Polyelectrolyte Doped Hollow Nanofibers for Positional Assembly of Bienzyme System for Cascade Reaction at O/W Interface. ACS Catal..

[218] Esmaeili A., Haseli M. (2017). Electrospinning of thermoplastic carboxymethyl cellulose/poly(ethylene oxide) nanofibers for use in drug-release systems. Mater. Sci. Eng. C.

[219] Zhu J., Huang W., Zhang Q., Ling S., Chen Y., Kaplan D.L. (2016). Aqueous-Based Coaxial Electrospinning of Genetically Engineered Silk Elastin Core-Shell Nanofibers. Materials.

[220] Lee B.-S., Yang H.-S., Yu W.-R. (2014). Fabrication of double-tubular carbon nanofibers using quadruple coaxial electrospinning. Nanotechnology.

[221] Zhou X., Fu J., Li Y., Li F. (2013). Nanomechanical mapping of glass fiber reinforced polymer composites using atomic force acoustic microscopy. J. Appl. Polym. Sci..

[222] Forward K.M., Flores A., Rutledge G.C. (2013). Production of core/shell fibers by electrospinning from a free surface. Chem. Eng. Sci..

[223] Qi H., Hu P., Xu J., Wang A. (2006). Encapsulation of Drug Reservoirs in Fibers by Emulsion Electrospinning: Morphology Characterization and Preliminary Release Assessment. Biomacromolecules.

[224] Nikmaram N., Roohinejad S., Hashemi S., Koubaa M., Barba F.J., Abbaspourrad A., Greiner R. (2017). Emulsion-based systems for fabrication of electrospun nanofibers: Food, pharmaceutical and biomedical applications. RSC Adv..

[225] Agarwal S., Greiner A. (2011). On the way to clean and safe electrospinning-green electrospinning: Emulsion and suspension electrospinning. Polym. Adv. Technol..

[226] Xu X., Yang L., Xu X., Wang X., Chen X., Liang Q., Zeng J., Jing X. (2005). Ultrafine medicated fibers electrospun from W/O emulsions. J. Control. Release.

[227] Bazilevsky A.V., Yarin A.L., Megaridis C.M. (2007). Co-electrospinning of Core−Shell Fibers Using a Single-Nozzle Technique. Langmuir.

[228] Yarin A. (2010). Coaxial electrospinning and emulsion electrospinning of core-shell fibers. Polym. Adv. Technol..

[229] Xu X., Zhuang X., Chen X., Wang X., Yang L., Jing X. (2006). Preparation of Core-Sheath Composite Nanofibers by Emulsion Electrospinning. Macromol. Rapid Commun..

[230] Crespy D., Friedemann K., Popa A.-M. (2012). Colloid-Electrospinning: Fabrication of Multicompartment Nanofibers by the Electrospinning of Organic or/and Inorganic Dispersions and Emulsions. Macromol. Rapid Commun..

[231] Rajzer I., Kurowska A., Jabłoński A., Jatteau S., Śliwka M., Ziąbka M., Menaszek E. (2018). Layered gelatin/PLLA scaffolds fabricated by electrospinning and 3D printing- for nasal cartilages and subchondral bone reconstruction. Mater. Des..

[232] Chiara G., Letizia F., Lorenzo F., Edoardo S., Diego S., Stefano S., Eriberto B., Barbara Z. (2012). Nanostructured Biomaterials for Tissue Engineered Bone Tissue Reconstruction. Int. J. Mol. Sci..

[233] Cao D., Wu Y.-P., Fu Z.-F., Tian Y., Li C.-J., Gao C.-Y., Chen Z.-L., Feng X.-Z. (2011). Cell adhesive and growth behavior on electrospun nanofibrous scaffolds by designed multifunctional composites. Colloids Surfaces B Biointerfaces.

[234] Yu N.Y.C., O’Brien C.A., Slapetova I., Whan R.M., Knothe Tate M.L. (2017). Live Tissue Imaging to Elucidate Mechanical Modulation of Stem Cell Niche Quiescence. Stem Cells Transl. Med..

[235] Lin Z., Fateh A., Salem D.M., Intini G. (2014). Periosteum: Biology and Applications in Craniofacial Bone Regeneration. J. Dent. Res..

[236] Squier C.A., Ghoneim S., Kremenak C.R. (1990). Ultrastructure of the periosteum from membrane bone. J. Anat..

[237] Sun F., Chen J., Jin S., Wang J., Man Y., Li J., Zou Q., Li Y., Zuo Y. (2018). Development of biomimetic trilayer fibrous membranes for guided bone regeneration. J. Mater. Chem. B.

[238] Liu F., Wang X., Chen T., Zhang N., Wei Q., Tian J., Wang Y., Ma C., Lu Y. (2020). Hydroxyapatite/silver electrospun fibers for anti-infection and osteoinduction. J. Adv. Res..

[239] Kareem M.M., Hodgkinson T., Sanchez M.S., Dalby M.J., Tanner K.E. (2019). Hybrid core–shell scaffolds for bone tissue engineering. Biomed. Mater..

[240] Tanner K.E. (2010). Bioactive composites for bone tissue engineering. Proc. Inst. Mech. Eng. Part H J. Eng. Med..

[241] Alam H.A., Dalgıç A.D., Tezcaner A., Ozen C., Keskin D. (2019). A comparative study of monoaxial and coaxial PCL/gelatin/Poloxamer 188 scaffolds for bone tissue engineering. Int. J. Polym. Mater. Polym. Biomater..

[242] Hunter R.L., Luo A.Z., Zhang R., Kozar R.A., Moore F.A. (2010). Poloxamer 188 inhibition of ischemia/reperfusion injury: Evidence for a novel anti-adhesive mechanism. Ann. Clin. Lab. Sci..

[243] Panda N., Bissoyi A., Pramanik K., Biswas A. (2015). Development of novel electrospun nanofibrous scaffold from P. ricini and A. mylitta silk fibroin blend with improved surface and biological properties. Mater. Sci. Eng. C.

[244] Zhang F., Zuo B.Q., Zhang H.X., Bai L. (2009). Studies of electrospun regenerated SF/TSF nanofibers. Polymer.

[245] Altman G.H., Horan R.L., Lu H.H., Moreau J., Martin I., Richmond J.C., Kaplan D.L. (2002). Silk matrix for tissue engineered anterior cruciate ligaments. Biomaterials.

[246] Shao W., He J., Sang F., Ding B., Chen L., Cui S., Li K., Han Q., Tan W. (2016). Coaxial electrospun aligned tussah silk fibroin nanostructured fiber scaffolds embedded with hydroxyapatite–tussah silk fibroin nanoparticles for bone tissue engineering. Mater. Sci. Eng. C.

[247] Choi Y., Cho S.Y., Heo S., Jin H.-J. (2013). Enhanced mechanical properties of silk fibroin-based composite plates for fractured bone healing. Fibers Polym..

[248] Nosar M.N., Salehi M., Ghorbani S., Pour Beiranvand S., Goodarzi A., Azami M. (2016). Characterization of wet-electrospun cellulose acetate based 3-dimensional scaffolds for skin tissue engineering applications: Influence of cellulose acetate concentration. Cellulose.

[249] Tao C., Zhang Y., Li B., Chen L. (2017). Hierarchical micro/submicrometer-scale structured scaffolds prepared via coaxial electrospinning for bone regeneration. J. Mater. Chem. B.

[250] Huang C., Dong J., Zhang Y., Chai S., Wang X., Kang S., Yu D., Wang P., Jiang Q. (2021). Gold Nanoparticles-Loaded Polyvinylpyrrolidone/Ethylcellulose Coaxial Electrospun Nanofibers with Enhanced Osteogenic Capability for Bone Tissue Regeneration. Mater. Des..

[251] Liang H., Xu X., Feng X., Ma L., Deng X., Wu S., Liu X., Yang C. (2019). Gold nanoparticles-loaded hydroxyapatite composites guide osteogenic differentiation of human mesenchymal stem cells through Wnt/β-catenin signaling pathway. Int. J. Nanomed..

[252] Zhang Y., Wang P., Mao H., Zhang Y., Zheng L., Yu P., Guo Z., Li L., Jiang Q. (2020). PEGylated gold nanoparticles promote osteogenic differentiation in in vitro and in vivo systems. Mater. Des..

[253] Pathmanapan S., Sekar M., Pandurangan A.K., Anandasadagopan S.K. (2022). Fabrication of Mesoporous Silica Nanoparticle–Incorporated Coaxial Nanofiber for Evaluating the In Vitro Osteogenic Potential. Appl. Biochem..

[254] Zhou X., Feng W., Qiu K., Chen L., Wang W., Nie W., Mo X., He C. (2015). BMP-2 Derived Peptide and Dexamethasone Incorporated Mesoporous Silica Nanoparticles for Enhanced Osteogenic Differentiation of Bone Mesenchymal Stem Cells. ACS Appl. Mater. Interfaces.

[255] Lu Y., Jiang H., Tu K., Wang L. (2009). Mild immobilization of diverse macromolecular bioactive agents onto multifunctional fibrous membranes prepared by coaxial electrospinning. Acta Biomater..

[256] Kuo S.M., Chang S.J., Niu G.C.-C., Lan C.-W., Cheng W.T., Yang C.Z. (2009). Guided tissue regeneration with use of β-TCP/chitosan composite membrane. J. Appl. Polym. Sci..

[257] Kharaziha M., Fathi M.H., Edris H., Nourbakhsh N., Talebi A., Salmanizadeh S. (2015). PCL-forsterite nanocomposite fibrous membranes for controlled release of dexamethasone. J. Mater. Sci. Mater. Electron..

[258] He M., Xue J., Geng H., Gu H., Chen D., Shi R., Zhang L. (2015). Fibrous guided tissue regeneration membrane loaded with anti-inflammatory agent prepared by coaxial electrospinning for the purpose of controlled release. Appl. Surf. Sci..

[259] Jose M.V., Thomas V., Johnson K.T., Dean D.R., Nyairo E. (2009). Aligned PLGA/HA nanofibrous nanocomposite scaffolds for bone tissue engineering. Acta Biomater..

[260] Tang Y., Chen L., Zhao K., Wu Z., Wang Y., Tan Q. (2016). Fabrication of PLGA/HA (core)-collagen/amoxicillin (shell) nanofiber membranes through coaxial electrospinning for guided tissue regeneration. Compos. Sci. Technol..

[261] Mahalingam S., Bayram C., Gultekinoglu M., Ulubayram K., Homer-Vanniasinkam S., Edirisinghe M. (2021). Co-Axial Gyro-Spinning of PCL/PVA/HA Core-Sheath Fibrous Scaffolds for Bone Tissue Engineering. Macromol. Biosci..

[262] Mahalingam S., Homer-Vanniasinkam S., Edirisinghe M. (2019). Novel pressurised gyration device for making core-sheath polymer fibres. Mater. Des..

[263] Yuan X., Zhang M., Wang Y., Zhao H., Sun D. (2019). Using co-axial electrospray deposition to eliminate burst release of simvastatin from microparticles and to enhance induced osteogenesis. J. Biomater. Sci. Polym. Ed..

[264] Wu S., Weng Z., Liu X., Yeung K., Chu P.K. (2014). Functionalized TiO_2_ Based Nanomaterials for Biomedical Applications. Adv. Funct. Mater..

[265] Kailasanathan C., Selvakumar N., Naidu V. (2012). Structure and properties of titania reinforced nano-hydroxyapatite/gelatin bio-composites for bone graft materials. Ceram. Int..

[266] Brammer K.S., Frandsen C.J., Jin S. (2012). TiO_2_ nanotubes for bone regeneration. Trends Biotechnol..

[267] Adhikari S.P., Pant H.R., Mousa H.M., Lee J., Kim H.J., Park C.H., Kim C.S. (2016). Synthesis of high porous electrospun hollow TiO_2_ nanofibers for bone tissue engineering application. J. Ind. Eng. Chem..

[268] Song P., Wang W., Li J., Cao S., Shi J. (2022). Self-assembly of hydroxyapatite around Ti_3_C_2_ MXene/gold nanorods for efficient remotely triggered drug delivery. Ceram. Int..

[269] Fu Y., Zhang J., Lin H., Mo A. (2020). 2D titanium carbide(MXene) nanosheets and 1D hydroxyapatite nanowires into free standing nanocomposite membrane: In vitro and in vivo evaluations for bone regeneration. Mater. Sci. Eng. C.

[270] Pogorielov M., Smyrnova K., Kyrylenko S., Gogotsi O., Zahorodna V., Pogrebnjak A. (2021). MXenes—A New Class of Two-Dimensional Materials: Structure, Properties and Potential Applications. Nanomaterials.

[271] Kyrylenko S., Gogotsi O., Baginskiy I., Balitskyi V., Zahorodna V., Husak Y., Yanko I., Pernakov M., Roshchupkin A., Lyndin M. (2022). MXene-Assisted Ablation of Cells with a Pulsed Near-Infrared Laser. ACS Appl. Mater. Interfaces.

[272] Sadat-Shojai M. (2016). Electrospun Polyhydroxybutyrate/Hydroxyapatite Nanohybrids: Microstructure and Bone Cell Response. J. Mater. Sci. Technol..

[273] Pal J., Sharma S., Sanwaria S., Kulshreshtha R., Nandan B., Srivastava R.K. (2014). Conducive 3D porous mesh of poly(ε-caprolactone) made via emulsion electrospinning. Polymer.

[274] Pal J., Singh S., Sharma S., Kulshreshtha R., Nandan B., Srivastava R.K. (2016). Emulsion electrospun composite matrices of poly(ε-caprolactone)-hydroxyapatite: Strategy for hydroxyapatite confinement and retention on fiber surface. Mater. Lett..

[275] Pal J., Wu D., Hakkarainen M., Srivastava R.K. (2017). The viscoelastic interaction between dispersed and continuous phase of PCL/HA-PVA oil-in-water emulsion uncovers the theoretical and experimental basis for fiber formation during emulsion electrospinning. Eur. Polym. J..

[276] Dai Y., Niu J., Liu J., Yin L., Xu J. (2010). In situ encapsulation of laccase in microfibers by emulsion electrospinning: Preparation, characterization, and application. Bioresour. Technol..

[277] Li X., Su Y., Liu S., Tan L., Mo X., Ramakrishna S. (2010). Encapsulation of proteins in poly(l-lactide-co-caprolactone) fibers by emulsion electrospinning. Colloids Surfaces B Biointerfaces.

[278] Yang Y., Li X., Cui W., Zhou S., Tan R., Wang C. (2007). Structural stability and release profiles of proteins from core-shell poly (DL-lactide) ultrafine fibers prepared by emulsion electrospinning. J. Biomed. Mater. Res. Part A.

[279] Srikar R., Yarin A.L., Megaridis C., Bazilevsky A.A.V., Kelley E. (2007). Desorption-Limited Mechanism of Release from Polymer Nanofibers. Langmuir.

[280] Tian L., Prabhakaran M.P., Ding X., Ramakrishna S. (2013). Biocompatibility evaluation of emulsion electrospun nanofibers using osteoblasts for bone tissue engineering. J. Biomater. Sci. Polym. Ed..

[281] Briggs T., Matos J., Collins G., Arinzeh T.L. (2015). Evaluating protein incorporation and release in electrospun composite scaffolds for bone tissue engineering applications. J. Biomed. Mater. Res. Part A.

[282] Okada M., Maeda H., Fujii S., Nakamura Y., Furuzono T. (2012). Formation of Pickering Emulsions Stabilized via Interaction between Nanoparticles Dispersed in Aqueous Phase and Polymer End Groups Dissolved in Oil Phase. Langmuir.

[283] Samanta A., Takkar S., Kulshreshtha R., Nandan B., Srivastava R.K. (2017). Hydroxyapatite stabilized pickering emulsions of poly(ε-caprolactone) and their composite electrospun scaffolds. Colloids Surf. A Physicochem. Eng. Asp..

[284] Pal J., Skrifvars M., Nandan B., Srivastava R.K. (2016). Electrospun composite matrices from tenside-free poly(ε-caprolactone)-grafted acrylic acid/hydroxyapatite oil-in-water emulsions. J. Mater. Sci..

[285] Abdal-hay A., Abbasi N., Gwiazda M., Hamlet S., Ivanovski S. (2018). Novel polycaprolactone/hydroxyapatite nanocomposite fibrous scaffolds by direct melt-electrospinning writing. Eur. Polym. J..

[286] Li X., Liu H., Wang J., Li C. (2012). Preparation and characterization of PLLA/nHA nonwoven mats via laser melt electrospinning. Mater. Lett..

[287] Wang Z., Wang H., Xiong J., Li J., Miao X., Lan X., Liu X., Wang W., Cai N., Tang Y. (2021). Fabrication and in vitro evaluation of PCL/gelatin hierarchical scaffolds based on melt electrospinning writing and solution electrospinning for bone regeneration. Mater. Sci. Eng. C.

